# Global, regional, and national prevalence of prostate cancer from 1990 to 2021: a trend and health inequality analyses

**DOI:** 10.3389/fpubh.2025.1595159

**Published:** 2025-06-11

**Authors:** Xiaohu Zhao, Shuchen Liu, Zhihui Zou, Chaozhao Liang

**Affiliations:** Department of Urology, The First Affiliated Hospital of Anhui Medical University, Hefei, China

**Keywords:** prostate cancer, prevalence, Global Burden of Disease study 2021, health inequality, national

## Abstract

**Background:**

Prostate cancer in men's health has become a significant driver of global disease burden, impacting aging populations worldwide. This study assesses its prevalence from 1990 to 2021 to reveal ongoing epidemiological trends and inform effective public health strategies.

**Methods:**

Prostate cancer prevalence estimates, including their 95% uncertainty intervals (UIs), were derived from the Global Burden of Disease (GBD) 2021 study. Then, temporal trends spanning the past 32 years were thoroughly analyzed using Joinpoint regression, with projections for the next 25 years made using the Bayesian Age-Period-Cohort (BAPC) model. Concurrently, disease trends were decomposed into components of population growth, aging, and epidemiological changes. Additionally, age-period-cohort (APC) models were also employed to explore the impact of age, time, and cohort effect on the relative risk of prostate cancer prevalence. And the cross-country inequalities in the prevalence of prostate cancer burden were meticulously evaluated through the Socio-Demographic Index (SDI), revealing significant disparities across socio-economic strata.

**Result:**

In 2021, over 10 million prostate cancer cases were recorded worldwide—a 188.85% increase from 3.6 million in 1990. The age-standardized prevalence rate (ASPR) rose at an estimated annual percentage change of 0.64% (95% UI: 0.47%−0.82%); Joinpoint regression revealed a steady increase in case numbers over 32 years, while the ASPR peaked and then slightly declined. Decomposition analysis showed population growth as the main driver (65.62%), with epidemiological changes and aging accounting for 17.97 and 16.41%, respectively. APC modeling indicated the highest relative risk around age 75—nearly 10 times that of the general population (RR: 9.99; 95% CI: 9.97–10.01). Projections through 2046 forecast a continued rise in both total cases and ASPR.

**Conclusions:**

As a major health concern among older adult men, the global prevalence of prostate cancer has risen steadily since 1990, with population growth identified as the primary driver. Moreover, SDI-related disparities across 204 countries and territories have widened over time. Finally, the APC model forecasts a continuous increase in prevalence over the next 25 years, underscoring the growing disease burden and the urgent need for more targeted and effective management strategies.

## Introduction

Prostate cancer is one of the most common cancers globally and, among men, it is the second most common cancer, which accounts a significant proportion of all cancer-related deaths (1–3). Specifically, prostate cancer accounted for ~1.3 million new cases globally in 2018 and claimed the lives of over 359,000 men, making it one of the most commonly diagnosed cancers in men (4). And, at the individual level, prostate cancer exerts a profound impact on men's health, with urinary symptoms such as frequent urination and difficulty urinating significantly impairing quality of life (5). Additionally, sexual dysfunction and mental health challenges, including anxiety and depression, are frequently observed among prostate cancer patients (6). This dual burden, encompassing both physical and emotional challenges, significantly erodes quality of life and places a profound emotional strain on families, often leading to heightened anxiety, depression, and a deep sense of helplessness within the household (7, 8).

Not only does prostate cancer have a profound impact on patients and their families, but its economic burden is substantial on both global and national scales. Globally, the economic cost of cancers, including prostate cancer, is projected to account for ~0.55% of the annual global gross domestic product (GDP) from 2020 to 2050, highlighting its significant impact on economies worldwide (9). In Sweden, the annual cost of prostate cancer, including healthcare expenses and productivity losses, is estimated at ~€1.1 billion, further underscoring its financial strain (10). Similarly, in the United Kingdom, prostate cancer treatment costs alone exceed £500 million annually, representing a considerable economic burden on the healthcare system (11).

However, the etiology of prostate cancer remains unclear, though various risk factors, including age, family history, dietary factors, and obesity, are believed to contribute to its development. Increasing age is one of the strongest risk factors, with prostate cancer incidence rising exponentially with age (1). Dietary factors, such as high intake of animal fat, have also been associated with increased risk (12). Additionally, obesity has been linked to more aggressive forms of prostate cancer (13). Therefore, in the absence of clear etiological factors, understanding its epidemiology becomes crucial in developing targeted management strategies.

The Global Burden of Disease (GBD) study provides comprehensive data on diseases, injuries, and risk factors worldwide, assessing their impact on different regions and populations by analyzing health data and offering indicators such as incidence, deaths, and disability-adjusted life years (DALYs) (14). Although existing studies have used GBD data to explore the epidemiological features of prostate cancer, they are either outdated or have overlooked the important epidemiological factor of prostate cancer prevalence (15–17). Specifically, the study conducted by Fazlollah and colleagues primarily focuses on the relationship between prostate cancer burden [prevalence, mortality, and disability-adjusted life years (DALY)] and the Human Development Index (HDI) in Asia. However, this study is limited to the disease burden in the Asian region and fails to provide insights into the global prostate cancer burden. Additionally, the study only addresses trends over the past 30 years and does not offer predictions for future disease burdens, thus providing no reference for future policy development (15). In contrast, more recent studies, such as those by Zhang et al. (17). and Chu et al. (18), address the global burden and socioeconomic factors, but still face significant limitations. Zhang et al. (17) provides a broad view of prostate cancer trends across socioeconomic strata from 1990 to 2019 but lacks projections beyond this period. Similarly, Chu et al. (18) offers a global analysis of prostate cancer burden, extending to 2021, but focuses primarily on age-period-cohort effects, without addressing key aspects of inequality or future projections. Additionally, with breakthroughs in prostate cancer treatments, including advancements in targeted therapies, immunotherapy, and minimally invasive surgical techniques, the survival rate of prostate cancer patients has been steadily increasing in recent years (19, 20). As the survival rate of prostate cancer patients increases, the disease characteristics are shifting toward a chronic, non-fatal condition, highlighting the urgent need to focus on its prevalence as a key aspect of the disease. Over the past few decades, there has been a notable and continuous increase in the global prevalence of prostate cancer. However, the important epidemiological indicator of the prevalence of prostate cancer has not received enough attention in previous studies, and there has been no dedicated research focusing on the changes in the prevalence of prostate cancer.

Based on the GBD database, we provide the most up-to-date and comprehensive assessment of global prostate cancer prevalence and forecast its prevalence for the next 25 years. Specifically, the primary objectives of this study are: (1) to analyze the trends in prostate cancer prevalence globally, regionally, and nationally over the past 32 years (1990–2021), focusing on variations across different Socio-Demographic Index (SDI) levels and examining how these trends have evolved over time, with particular attention to disparities between high and low SDI countries. (2) To identify and investigate the key drivers behind the changes in prostate cancer prevalence, emphasizing the roles of population aging, demographic growth, and epidemiological shifts, while also exploring cross-country inequalities in disease burden across varying socio-economic conditions. (3) To forecast the future burden of prostate cancer, estimating both the number of prevalent cases and the rate of increase in prevalence through to the year 2046, taking into account demographic changes, medical advancements, and global health disparities.

Building upon the aforementioned three objectives, this study facilitates a more comprehensive understanding of the global burden associated with prostate cancer prevalence. Of particular concern is the continuous increase in prevalence observed in recent years, with projections indicating further growth in the future. This upward trend presents considerable challenges to healthcare systems across nations and underscores the urgency of strategic planning in health resource allocation and policymaking. In countries with different levels of the Socio-Demographic Index (SDI), disparities in healthcare accessibility, screening coverage, and public health awareness may contribute to widening inequalities in disease burden. Therefore, a thorough understanding of the evolving patterns of prostate cancer prevalence is essential for guiding evidence-based health planning, optimizing resource allocation, and formulating tailored screening and intervention strategies appropriate to specific national contexts.

## Methods

### Data source and disease burden estimation

The Global Burden of Disease (GBD) Study meticulously assessed health loss across 204 countries and territories utilizing contemporary epidemiological data and rigorous standardized methodologies, identifying 369 diseases, injuries, and 87 risk factors as pivotal contributors to health deterioration (21, 22). The case numbers and age-standardized prevalence rates (ASPR) of prostate cancer from 1990 to 2021 were downloaded from the GBD website (http://ghdx.healthdata.org/gbd-results-tool), accessed on December 20, 2024. The case number and age-standardized prevalence rate (ASPR) were directly obtained from the “all ages” and “age-standardized” age groups in the GBD 2021 database without any further processing. Additionally, estimates and their 95% uncertainty intervals (UI) for prevalence were also extracted. Simultaneously, to further investigate the relationship between disease prevalence and socio-economic development, the Socio-Demographic Index (SDI)—which encompasses education, economic status, and fertility levels—was also extracted. The institutional review board deemed that the study did not require approval, as it utilized publicly available data. All methods were conducted in accordance with relevant guidelines and regulations.

### Temporal trend analysis

The age-standardized prevalence rate (ASPR) and their estimated annual percentage change (EAPC) were utilized to quantify the prevalence trends of prostate cancer. EAPC is a summary measure commonly used to assess the trend in the age-standardized rate (ASR) over a specific time interval. A regression line was fitted to the natural logarithm of the age-standardized rate (ASR) values, represented by the equation (*y* = α + β*x* + ϵ), when *y* = ln (ASR) and *x* = calendar year. The EAPC was calculated as 100 × [exp (β)−1], with its 95% confidence interval (CI) derived from the linear regression model (23, 24). ASPR is classified as follows: (1) increasing if the 95% confidence interval (CI) of the EAPC is entirely above zero; (2) decreasing if the 95% CI of the EAPC is entirely below zero; and (3) stable if the 95% CI of the EAPC includes zero.

Joinpoint regression analysis was applied to divide the temporal trends in disease progression into distinct segments, allowing the identification of statistically significant inflection points where the direction or magnitude of the trends shifted (25, 26). Joinpoint regression models (Joinpoint Regression Software, Version 5.1.0.0—April 2024, National Cancer Institute), a sophisticated set of statistical tools, were used to analyze temporal trends in the burden of prostate cancer prevalence. The analysis begins by identifying statistically significant changes in trends using Joinpoint regression, segmenting the data into distinct intervals to highlight critical inflection points. Using Joinpoint regression software, the analysis begins with a zero-joinpoint model, progressively testing up to nine joinpoints, employing Monte Carlo sampling of permuted datasets to compare the ratio of sums of squared errors between the null (simpler) and alternative (more complex) models and derive a *p*-value (27, 28). To control the overall type I error rate across these multiple comparisons, a Bonferroni correction is applied, and the iteration halts when the addition of another joinpoint no longer yields a statistically significant improvement, thus yielding the most parsimonious yet adequately fitting segmented trend model. With the Joinpoint regression model, the annual percentage change (APC) was calculated for each identified segment, offering a widely used metric to quantify the annual rate of change in specific health indicators. A positive APC indicates an upward trend, while a negative APC reflects a downward trend, with statistical significance determined at *p* < 0.05 (29).

### Age-period-cohort analysis

Trends in prostate cancer prevalence were examined through the effects of age, period, and birth cohort, providing a comprehensive analysis that disentangled the distinct contributions of these factors and revealed how demographic shifts and temporal changes collectively shaped disease progression over time (30). To achieve this, a preliminary analysis was conducted to assess potential two-factor interactions among age, period, and birth cohort effects (Supplementary Figure S1). The complex interdependence of these factors presents significant challenges in isolating their distinct contributions to prevalence risks, necessitating the use of advanced modeling techniques to unravel overlapping influences. This complex interplay highlights the necessity of a sophisticated analytical framework capable of accurately identifying the individual effects of age, period, and cohort. Therefore, the age-period-cohort (APC) model was adopted, incorporating these three dimensions to unravel their overlapping influences on the trends of prostate cancer prevalence (31). The APC model uses a logarithmic Poisson framework applied to observed rates on a Lexis diagram, facilitating the quantification of the additive effects of age, period, and cohort. It is widely adopted in descriptive epidemiological studies due to its significant advantages in analyzing complex disease trends (31, 32). Accordingly, the APC analysis in this study was conducted using the APC package in Stata. To address the inherent multicollinearity, or identification problem, among age, period, and cohort effects, the model employs linear constraint-based methods, such as setting the mean of one set of parameters (e.g., period or cohort) to zero or applying smoothing constraints like natural splines, thereby ensuring model identifiability and enabling robust estimation of individual effects. For the APC analysis, data from the GBD database were recoded into consecutive 5-year age groups (20–24, 25–29, …, 90–94, 95+ years), 5-year time periods spanning from 1990 to 2019 (1990–1994, 1995–1999, …, 2010–2014, 2015–2019), and corresponding 5-year birth cohorts (1895–1899, 1900–1904, …, 1990–1994, 1995–1999). This systematic recoding facilitated the estimation of the net effects of age, period, and cohort on the prevalence associated with prostate cancer, ensuring robust analytical outcomes. The APC model provides regression coefficients for each factor, which are transformed into relative risks (RR) using the formula RR = exp (coefficient). These RR values represent the risk ratio of a specific age, period, or cohort compared to the reference level. A value >1 indicates increased risk, while a value below 1 indicates reduced risk relative to the average across all groups (33, 34). In our study, we implemented the age-period cohort (APC) model, using the APC package in Stata (version 17.0) to analyze prostate cancer prevalence data.

### Decomposition analysis

Decomposition analysis was used to visually demonstrate the role of the three factors (aging, population growth, and epidemiological changes) driving changes in the prevalence of prostate cancer from 1990 to 2021. Specifically, we adopted a weighted average decomposition analysis, which attributes the total variation in health outcomes to these three components in a balanced and interpretable manner. The total burden at each time point was defined as $Y=\underset{i}{\sum }P_{i\;}\times r_{i\;}$, where *P*_i_ represents the population in age group *i*, and *r*_i_ is the corresponding age-specific rate (e.g., incidence, deaths, or DALY rate). The change in overall burden between 1990 and 2021 is given by $\Delta Y=Y_{2021}-Y_{1990}=\underset{i}{\sum }\left(P_{2021,i}\cdot r_{2021,i}-P_{1990,i}\cdot r_{1990,i}\right)$. This change was then decomposed into three distinct effects. The population growth effect (PG) accounts for changes in total population size while holding the age structure and disease rates constant, and is calculated as $PG=\underset{i}{\sum }\left(P_{2021,i}-P_{1990,i}\right)\cdot$${\bar{r}}_{\text{i}}$, where ${\bar{r}}_{\text{i}}$ $=\frac{r_{1990,i}+r_{2021,i}}{2}$. The population aging effect (PA) captures shifts in age structure under fixed rates and total population, and is estimated by $PA=\underset{i}{\sum }\left(\frac{P_{2021,i}}{P_{2021}}-\frac{P_{1990,i}}{P_{1990}}\right)\;P_{2021}\cdot r_{1990,i}$, where the term in parentheses reflects the relative change in the proportion of each age group. The epidemiological effect (EE) reflects changes in age-specific rates due to risk factors or healthcare improvements, computed as $EE=\underset{i}{\sum }P_{2021}\cdot \left(r_{2021,i}-r_{1990,i}\right)$. To avoid bias in favor of either year, we applied a weighted average approach, using ${\bar{r}}_{\text{i}}$ $=\frac{r_{2021,i}+r_{1990,i}}{2}$, $P_{\text{i}}=\frac{P_{1990,i}+P_{2021,i}}{2}$, ensuring both time points contributed equally to the analysis (35). To further explore the regional variations in the impact of these three factors on prostate cancer prevalence, data were analyzed not only globally but also across five SDI regions and 21 GBD regions.

### Cross-country inequalities analysis

Cross-country inequalities analysis was used to examine disparities in health outcomes and prevalence across different nations, highlighting variations in disease burden on the impact of socio-economic factors. And, the Slope Index of Inequality (SII) and Concentration Index (CI) were used to quantify the Socio-Demographic Index (SDI)-related inequalities in prostate cancer burden across countries, based on extensive prior research from the Global Burden of Disease (GBD) studies (22, 36–40). And the calculation formulas are as follows: SII = β_highest_ – β_lowest_. CI = 2 ^*^ Σ (*p*_j_^*^ *y*_j_^*^ *R*_j_) – 1. where *p*_j_ is the population share of group *j*, *y*_j_ is the health indicator value for group *j*, and *R*_j_ is the relative fractional rank of group *j* in the socioeconomic distribution (41, 42). Specifically, SII is used to assess the extent of health inequality across different socioeconomic gradients such as income or education levels, which quantifies the absolute difference in health outcomes (such as disease burden or mortality rates) between individuals at the top and bottom ends of the socioeconomic spectrum. CI quantifies the extent to which health outcomes are concentrated among affluent or impoverished populations. A positive CI indicates that the health variable is more concentrated among affluent groups, while a negative CI indicates higher concentration among impoverished groups. The closer the CI is to zero, the more equitable the distribution of the health variable across socioeconomic groups.

### Predictive analysis

To forecast the number and rate of prevalence from 2022 to 2046, we employed a linear log age–period–cohort model using the NORDPRED package in R software, which effectively accounts for exponential growth trends while constraining projections to linear trends (43, 44). The NORDPRED package utilizes existing prostate cancer prevalence data, world standard population proportions, and the United Nations' population projections to forecast the future disease burden of prostate cancer (43). Additionally, the projection uses the average values of data from adjacent 5-year periods, so the results reflect the average level for each 5-year interval. In this study, the NORDPRED model yielded highly accurate forecasting results, as evidenced by a residual sum of squares (RSS) of 3,969,281,253, a mean squared error (MSE) of 49,616,016, a root mean square error (RMSE) of 7,043.864, and a pseudo *R*^2^ based on variance of 0.9998494. These metrics indicate an excellent model fit and predictive performance, consistent with previous applications of NORDPRED in epidemiological projections.

## Result

### Global and regional burden

Globally, the case number of prostate cancer increased from 3,596,220 (95% UI: 3,445,437–3,705,436) in 1990 to 10,387,521 (95% UI: 9,705,680–10,904,400) in 2021, an increase of 188.85%. In addition, the ASPR for prostate cancer was 94.17 (95% UI: 90.06–97.12) in 1990, 119.41 (95% UI: 111.69–125.27) in 2021, and 0.64% (95% CI: 0.47–0.82) in EAPC, which is increased. The ASPR varies widely from SDI regions, with the highest ASPR in high SDI region, 228.22 in 1990 (95% UI: 220.24–234.89) and 283.9 in 2021 (95% UI: 268.73–295.71). The lowest ASPR was 18.56 (95% UI: 15.32–20.68) in 1990 in the low-middle SDI region and 34.16 (95% UI: 22.88–41.86) in 2021 in the low SDI region. ASPR increased in the all five SDI regions, and low-middle SDI regions experienced the greatest increase (EAPC: 2.45 95% CI: 2.31–2.59), while high SDI regions saw the smallest growth (EAPC: 0.57 95% CI: 0.36–0.78). In terms of the 21 GBD regions, high-income North America (ASPR: 444.75 95% UI: 427.13–459.43) and Australasia (ASPR: 430.06 95% UI: 373.23–496.92) recorded the highest ASPR in 1990 and 2021, respectively. Meanwhile, from 1990 to 2021, Eastern Europe (EAPC: 4.08 95% CI: 3.83–4.33) recorded the highest growth rate, while high-income North America (EAPC: −0.53 95% CI: −0.66 to −0.41), which once had the highest prevalence, experienced a decline in prevalence against the backdrop of rising global rates (Table 1).

**Table 1:** The case number and ASPR of prostate cancer in 1990 and 2021 and its temporal trends from 1990 to 2021.

**Location**	**1990**		**2021**		**1990–2021 EAPC (95% UI)**
	**Case number (95% UI)**	**ASPR (95% UI)**	**Case number (95% UI)**	**ASPR (95% UI)**	
Global	3,596,220 (3,445,437, 3,705,436)	94.17 (90.06, 97.12)	10,387,521 (9,705,680, 10,904,400)	119.41 (111.69, 125.27)	0.64 (0.47, 0.82)
**SDI**					
High SDI	2,607,410 (2,513,878, 2,683,485)	228.22 (220.24, 234.89)	5,987,872 (5,660,940, 6,245,000)	283.9 (268.73, 295.71)	0.57 (0.36, 0.78)
High-middle SDI	5,70,034 (540,995, 596,896)	57.13 (54.24, 59.74)	2,060,335 (1,849,681, 2,229,490)	100.93 (90.58, 109.12)	1.85 (1.63, 2.07)
Middle SDI	265,209 (233,036, 285,412)	27.48 (24.38, 29.46)	1,591,026 (1,374,887, 1,786,559)	58.84 (50.94, 65.91)	2.27 (2.05, 2.49)
Low-middle SDI	104,284 (85,175, 116,315)	18.56 (15.32, 20.68)	570,721 (479,428, 656,395)	40.65 (34.41, 46.58)	2.45 (2.31, 2.59)
Low SDI	45,357 (31,012, 56,284)	21.74 (15.04, 26.78)	163,701 (108,548, 202,254)	34.16 (22.88, 41.86)	1.42 (1.32, 1.52)
**Region**					
Andean Latin America	10,504 (8,360, 13,160)	54.81 (43.78, 68.69)	75,652 (55,386, 104,360)	130.69 (95.71, 179.84)	2.75 (2.51, 2.99)
Australasia	85,970 (79,054, 91,673)	350.52 (322, 373.41)	235,141 (204,068, 271,001)	430.06 (373.23, 496.92)	0.25 (−0.49, 0.99)
Caribbean	45,218 (41,833, 49,265)	174.85 (162, 190.32)	163,762 (137,670, 189,250)	303.91 (255.55, 351.25)	1.71 (1.50, 1.93)
Central Asia	10,958 (10,256, 11,668)	23.09 (21.57, 24.57)	31,230 (28,034, 34,704)	38.24 (34.52, 42.41)	2.43 (2.13, 2.73)
Central Europe	83,362 (78,627, 88,281)	54.13 (51.06, 57.35)	338,084 (306,991, 370,397)	145.93 (132.39, 159.9)	3.46 (3.18, 3.73)
Central Latin America	87,911 (83,466, 91,295)	111.53 (105.96, 115.88)	558,239 (479,996, 640,213)	225.12 (193.85, 257.82)	1.81 (1.43, 2.18)
Central Sub-Saharan Africa	5,017 (3,284, 6,624)	26.22 (17.54, 33.94)	20,514 (12,801, 27,625)	40.85 (25.53, 54.85)	1.52 (1.19, 1.86)
East Asia	79,696 (60,406, 97,784)	9.4 (7.24, 11.5)	692,464 (512,655, 915,807)	30.46 (22.6, 40.16)	3.88 (3.69, 4.07)
Eastern Europe	147,971 (140,097, 155,338)	50.73 (48.03, 53.26)	571,270 (512,390, 625,712)	155.83 (139.98, 170.39)	4.08 (3.83, 4.33)
Eastern Sub-Saharan Africa	19,182 (12,089, 24,214)	27.56 (17.71, 34.47)	70,410 (45,744, 90,459)	43.88 (29.17, 55.64)	1.49 (1.38, 1.60)
High-income Asia Pacific	79,023 (74,683, 83,292)	40.14 (37.91, 42.29)	534,447 (475,300, 586,962)	106.28 (94.54, 117.09)	3.52 (2.93, 4.11)
High-income North America	1,608,512 (1,545,488, 1,663,299)	444.75 (427.13, 459.43)	2,829,393 (2,698,233, 2,946,883)	421.7 (402.18, 439.12)	−0.53 (−0.66, −0.41)
North Africa and Middle East	52,612 (39,260, 62,723)	33.06 (25.32, 39.6)	432,155 (310,010, 524,900)	100.2 (72.35, 121.2)	3.85 (3.71, 3.98)
Oceania	909 (648, 1,213)	35.84 (26.15, 46.73)	3,395 (2,209, 4,627)	52.1 (34.75, 70.85)	1.29 (1.25, 1.33)
South Asia	46,419 (33,953, 555,52)	8.8 (6.52, 10.49)	262,891 (213,984, 350,659)	17.98 (14.68, 23.84)	2.05 (1.88, 2.23)
Southeast Asia	42,332 (30,155, 48,704)	17.89 (12.89, 20.54)	274,478 (182,878, 336,698)	43.04 (28.93, 52.55)	2.87 (2.82, 2.92)
Southern Latin America	34,783 (31,406, 38,535)	74.13 (67.12, 82.09)	118,558 (101,797, 136,410)	133.58 (114.85, 153.44)	1.90 (1.50, 2.30)
Southern Sub-Saharan Africa	15,223 (10,870, 20,565)	59.07 (42.48, 78.74)	58,562 (44,816, 70,006)	101.66 (77.97, 120.87)	1.93 (1.81, 2.05)
Tropical Latin America	58,447 (55,455, 61,317)	66.09 (62.68, 69.28)	311,471 (292,602, 329,482)	120.6 (113.24, 127.61)	1.61 (1.21, 2.01)
Western Europe	1,047,294 (1,005,897, 1,095,046)	173.57 (166.61, 181.49)	2,670,002 (2,459,710, 2,848,675)	289.08 (266.84, 309.01)	1.59 (1.15, 2.03)
Western Sub-Saharan Africa	34,876 (20,321, 45,889)	42.41 (25.2, 55.27)	135,403 (71,247, 184,783)	75.36 (40.52, 101.49)	2.00 (1.93, 2.07)

ASPR, age-standardized prevalence rate; EAPC, estimated annual percentage change; SDI, Socio-Demographic Index; UI, uncertainty interval; CI, confidence interval.

### National burden

**Figure 1:**
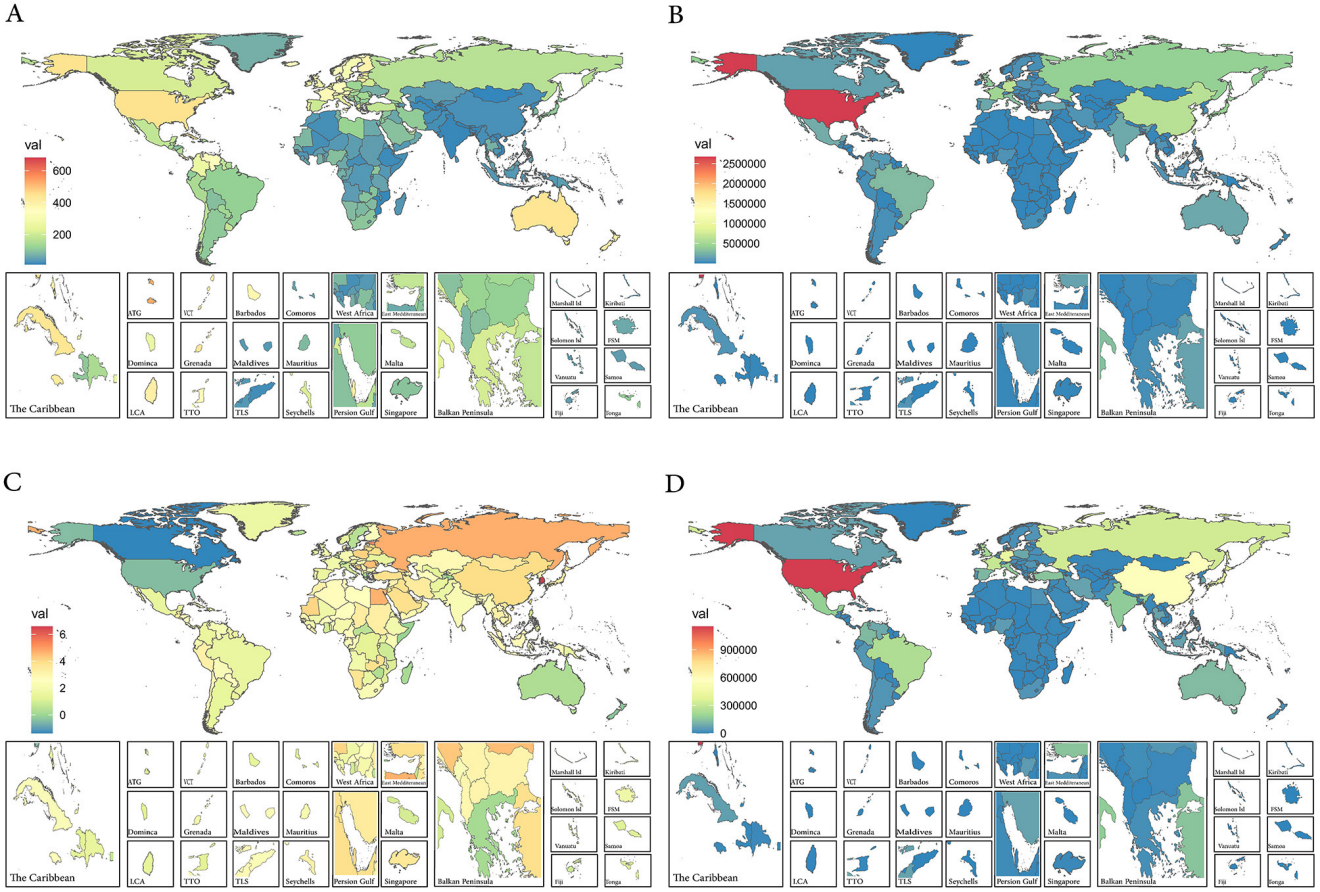
**(A)** The ASPR of prostate cancer in 2021. **(B)** The case number of prostate cancer in 2021. **(C)** The EAPC of ASPR of prostate cancer from 1990 to 2021. **(D)** The case number changes of prostate cancer from 1990 to 2021. ASPR, age-standardized prevalence rate; EAPC, estimated annual percentage change.

As for the 204 countries and territories, the United States had the highest prevalence number of prostate cancer patients globally in both 1990 (Case number: 1,522,355 95% UI: 1,465,187–1,576,800) and 2021 (Case number: 2,671,779 95% UI: 2,547, 383–2,786,354; Supplementary Table S1 and Figure 1B). Not only the most cases, the United States' ASPR was also the highest in the world in 1990 (ASPR: 463.92 95% UI: 447.23–479.53). By 2021, Bermuda had replaced it as the country with the highest prostate cancer prevalence in the world (ASPR: 680.35 95%UI: 537.53–855.07; Supplementary Table S1 and Figure 1A). From 1990 to 2021, South Korea (EAPC: 6.57 95% CI: 5.85–7.30) became the country with the fastest increase in prevalence globally, while the United States saw the largest increase in the prevalence numbers (Supplementary Table S1 and Figures 1C, D).

### Joinpoint regression analysis

**Figure 2:**
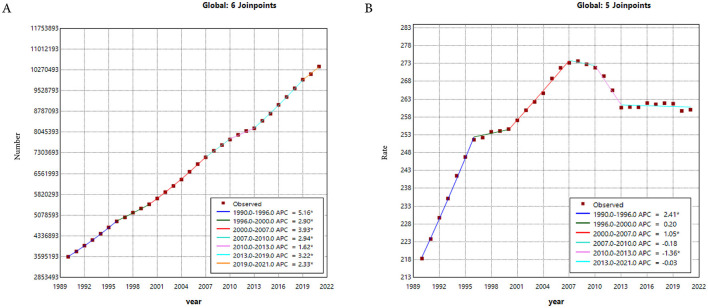
**(A)** The Joinpoint regression analysis on the case number of prevalence. **(B)** The Joinpoint regression analysis on the ASR of prevalence; of prostate cancer globally. ASR, age-standardized rate.

Between 1990 and 2021, prostate cancer prevalence showed consistently dramatic increases, with the most rapid increases observed during 1990–1996, 2000–2007, and 2013–2019, respectively (Figure 2A). Regarding the ASPR, it showed an upward trend from 1990 to 2007, followed by a decline from 2007 to 2021. Specifically, the trend for the ASPR experienced five joinpoints of slight increases in 1990–1996, 2000–2007, and 2010–2013, and no changes in 1996–2000, 2007–2010, and 2013–2021 (Figure 2B).

### Age-period-cohort analysis

**Figure 3:**
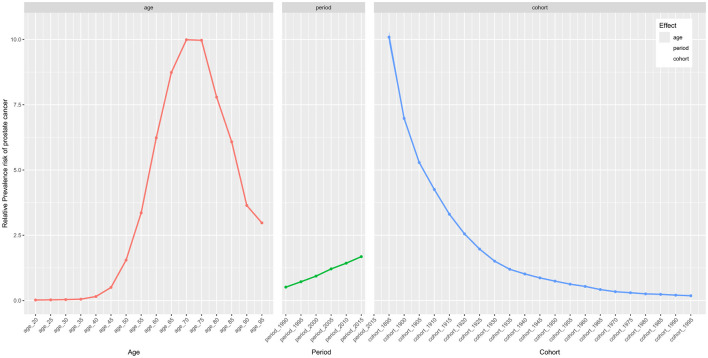
The effects of age, period, and birth cohort on the relative risk of prostate cancer prevalence.

Thanks to the APC analysis, we are able to control for two of the factors—age, period, and cohort—allowing us to explore the impact of the remaining factor on the relative prevalence risk. Regarding the age effect, it had a remarkable impact on prostate cancer prevalence, with the highest risk observed between the ages of 70–79 (Figure 3 and Table 2). Furthermore, we found that the risk of prostate cancer prevalence is higher in individuals over 50, indicating the need for focused prostate cancer risk management in older populations. Additionally, the period and cohort effects were found to significantly impact the risk of prostate cancer prevalence, with period effects causing a gradual increase in risk over time, while cohort effects showed a decrease yearly.

**Table 2:** RRs of prostate cancer prevalence due to age, period, and birth cohort effects.

**Factor**	**Prevalence**	
	**RR (95% CI)**	***p*** **-Value**
**Age (years)**		
20–24	0.020 (0.019, 0.020)	< 0.001
25–29	0.025 (0.025, 0.026)	< 0.001
30–34	0.036 (0.035, 0.036)	< 0.001
35–39	0.051 (0.051, 0.052)	< 0.001
40–44	0.152 (0.151, 0.153)	< 0.001
45–49	0.501 (0.499, 0.504)	< 0.001
50–54	1.547 (1.541, 1.554)	< 0.001
55–59	3.357 (3.346, 3.369)	< 0.001
60–64	6.233 (6.216, 6.250)	< 0.001
65–69	8.736 (8.716, 8.756)	< 0.001
70–74	9.990 (9.970, 10.010)	< 0.001
75–79	9.969 (9.949, 9.989)	< 0.001
80–84	7.791 (7.773, 7.809)	< 0.001
85–89	6.079 (6.061, 6.097)	< 0.001
90–94	3.641 (3.625, 3.656)	< 0.001
95 plus	2.973 (2.953, 2.994)	< 0.001
**Period**		
1990	0.513 (0.512, 0.514)	< 0.001
1995	0.718 (0.717, 0.719)	< 0.001
2000	0.934 (0.934, 0.935)	< 0.001
2005	1.210 (1.209, 1.211)	< 0.001
2010	1.429 (1.428, 1.431)	< 0.001
2015	1.680 (1.677, 1.684)	< 0.001
**Birth cohort**		
1895–1899	10.089 (9.873, 10.310)	< 0.001
1900–1904	6.976 (6.904, 7.049)	< 0.001
1905–1909	5.287 (5.255, 5.319)	< 0.001
1910–1914	4.255 (4.235, 4.275)	< 0.001
1915–1919	3.306 (3.293, 3.319)	< 0.001
1920–1924	2.554 (2.546, 2.563)	< 0.001
1925–1929	1.971 (1.965, 1.978)	< 0.001
1930–1934	1.508 (1.503, 1.512)	< 0.001
1935–1939	1.197 (1.193, 1.201)	< 0.001
1940–1944	1.018 (1.014, 1.021)	< 0.001
1945–1949	0.867 (0.863, 0.870)	< 0.001
1950–1954	0.742 (0.739, 0.746)	< 0.001
1955–1959	0.626 (0.623, 0.630)	< 0.001
1960–1964	0.540 (0.537, 0.543)	< 0.001
1965–1969	0.420 (0.417, 0.422)	< 0.001
1970–1974	0.338 (0.335, 0.341)	< 0.001
1975–1979	0.298 (0.295, 0.301)	< 0.001
1980–1984	0.254 (0.251, 0.258)	< 0.001
1985–1989	0.237 (0.233, 0.241)	< 0.001
1990–1994	0.205 (0.201, 0.210)	< 0.001
1995–1999	0.182 (0.175, 0.189)	< 0.001

RR, relative risk.

### Decomposition analysis

**Figure 4:**
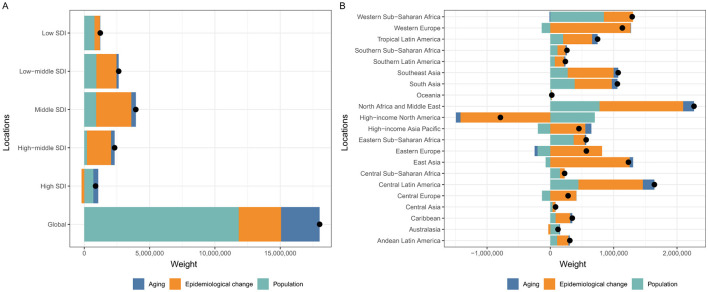
Changes in prevalence of prostate cancer according to population-level determinants including aging, population growth and epidemiological change from 1990 to 2021 at the global level and by SDI quintiles **(A)** and 21 regions **(B)**. SDI, Socio-Demographic Index.

A decomposition analysis was developed to explore the role of the three factors (aging, population growth, and epidemiological change) driving changes in the prevalence of prostate cancer. Globally, population growth contributed the most, accounting for 65.62% of the increase in prostate cancer prevalence between 1990 and 2021, while aging and epidemiological changes contributed 16.41 and 17.97%, respectively. For the five SDI regions, population growth was most pronounced in the high SDI regions, accounting for 80.47%, while in the other four SDI regions, the primary influencing factor was epidemiological change (Supplementary Table S2 and Figure 4A). Additionally, a similar decomposition analysis was conducted for the 21 GBD regions. Remarkably, epidemiological change was the dominant factor driving the decline in prostate cancer prevalence in the high–income North America region, despite a global increase (Figure 4B).

### Cross-country inequality analysis

**Figure 5:**
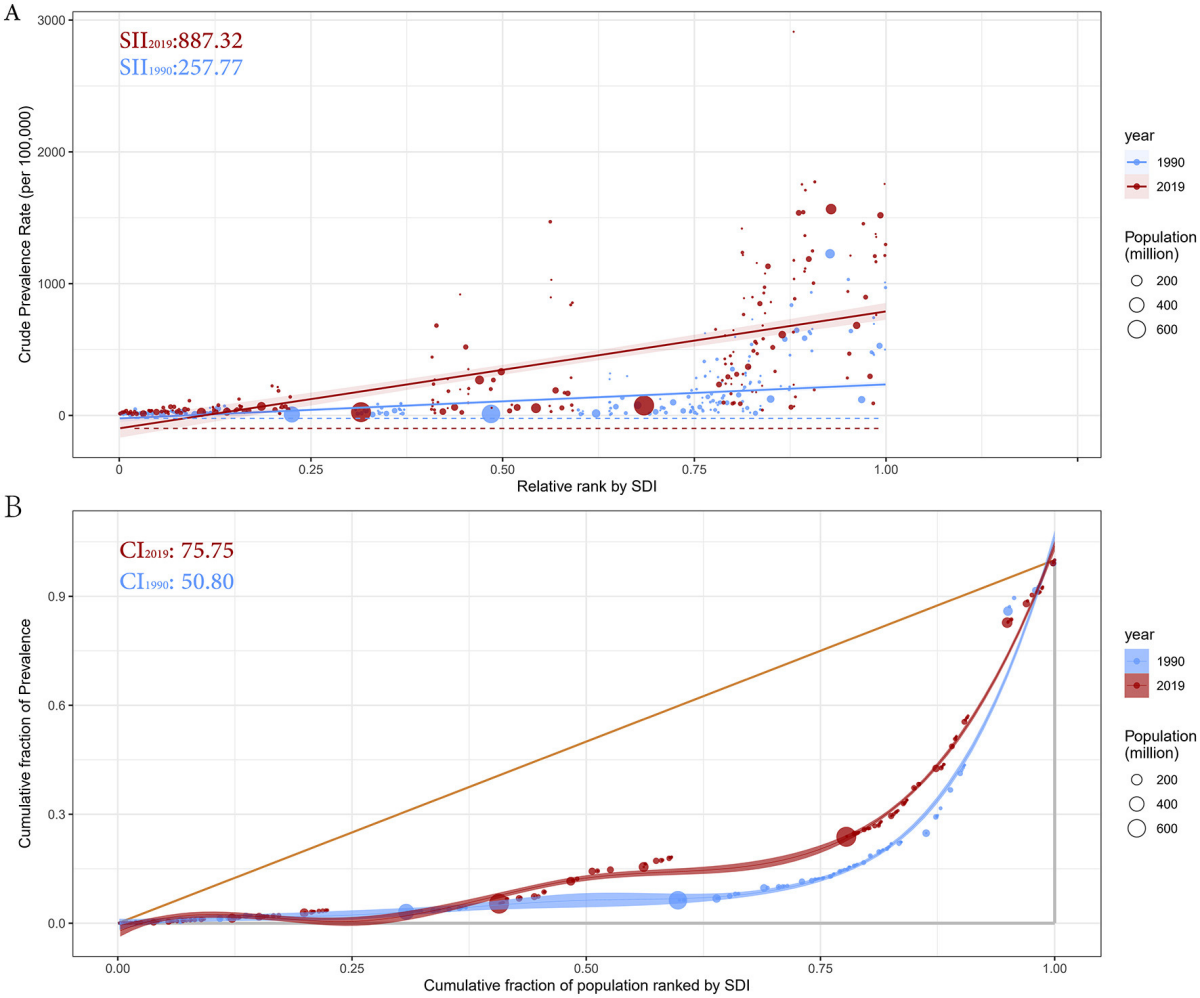
Health inequality regression curves **(A)** and concentration curves **(B)** for the prevalence of prostate cancer from 1990 to 2019 across the world. SII, Slope Index of Inequality; CI, Concentration Index.

Remarkable absolute and relative SDI-related inequalities in the prevalence of prostate cancer were detected, with this imbalance intensifying over time (Figure 5). In 1990, the SII was 257.77 (95% CI: 204.51–311.03), indicating that the disease prevalence in the highest SDI country was 257.77 per 100,000 higher than in the lowest SDI country. By 2019, this gap had more than tripled, reaching 887.32 (95% CI: 746.61–1028.03) per 100,000 (Figure 5A). Meanwhile, the CI value increased from 50.80 (95% CI: 39.72–59.41) in 1990 to 75.75 (95% CI: 67.20–84.74) in 2019, further indicating significant relative health inequality between countries (Figure 5B). In summary, significant health inequalities in prostate cancer prevalence exist across countries.

### Predicted trends

**Figure 6:**
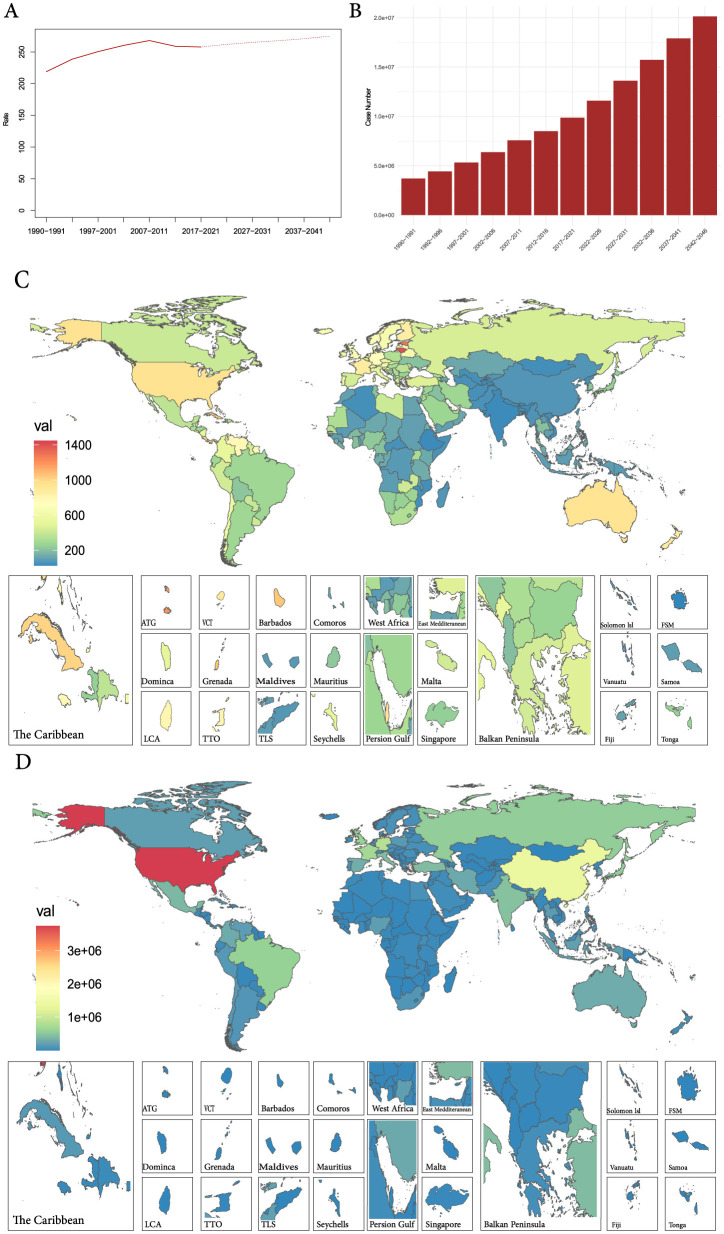
**(A)** The global change trends of ASPR of prostate cancer from 1990 to 2021, and its predicted trends between 2022 and 2046. **(B)** The global change trends of case number of prostate cancer from 1990 to 2019, and its predicted trends between 2022 and 2046. **(C)** The predicted ASPR of prostate cancer in 2042–2046 among 192 countries and territories. **(D)** The predicted case number of prostate cancer in 2042–2046 among 192 countries and territories. ASPR, age-standardized prevalence rate.

Using the linear log age-period-cohort model, the global ASPR and case numbers for prostate cancer are projected for 2022–2046, revealing an upward trend for both (Figures 6A, B and Supplementary Table S3). As for the 204 countries and territories, the United States still has the highest case number globally, but the prevalence of prostate cancer in most countries will be challenging (Figures 6C, D and Supplementary Table S4).

## Discussion

Prostate cancer is the second most common solid tumor in men and the fifth cause of cancer mortality (2). Currently, prostate cancer and its treatment have a significant impact on men's health, often leading to urinary incontinence and erectile dysfunction, which greatly affect the quality of patients life (45, 46). These issues can also lead to psychological problems such as anxiety, depression, and decreased self-esteem, which greatly affect the mental health of patients (46). Prostate cancer not only poses significant health challenges for patients but also imposes a substantial economic burden on both the nation and individual families. Research has indicated that the productivity losses caused by prostate cancer amount to up to $7.2 billion annually in the United States (47). For families, especially those with lower incomes, the economic burden is even more severe, patients may face higher out-of-pocket expenses, and some may struggle to afford the costs associated with their care (48). Therefore, placing greater emphasis on the early detection and treatment of prostate cancer will not only alleviate patient suffering but also help reduce the economic burden on the nation. This requires us to put significant effort into disease prevention, with a primary focus on understanding the epidemiological characteristics of the disease.

When discussing disease prevention, the importance of risk factors cannot be overstated. Currently, age, family history, and genetic predisposition/germline variants are recognized as significant risk factors for prostate cancer (49–52). However, since these factors cannot be effectively prevented or altered, research is increasingly focusing on modifiable risk factors. And, itis confirmed that factors such as physical activity and sleep, dietary intake, sexual activity, infectious agents, and marital status are closely related to the occurrence and progression of prostate cancer (1). While prevention strategies targeting these risk factors can be effective on an individual level, there is a pressing need for high-quality epidemiological evidence at the global or national level to develop appropriate regional policies.

In 2021, there were over 10 million prostate cancer patients globally, with more than half of them concentrated in high-SDI regions. This concentration of prostate cancer in high-SDI regions is not only evident in prevalence but also observed in studies focusing on incidence, deaths, and DALYs (17, 53). Not surprisingly, the ASPR in high-SDI region is the highest among the five SDI regions, while the EAPC is the lowest, likely due to the higher baseline ASPR in 1990. It is particularly noteworthy that, in 2021, the number of prostate cancer patients in the United States reached 3.6 million, accounting for more than half of the cases in high-SDI regions. This phenomenon reflects the high incidence and low mortality rate of prostate cancer in the United States, aligning with previous research findings (17, 54). Additionally, among the 21 GBD regions, the prevalence of prostate cancer in the high-income North America region has shown a declining trend over the past 32 years, in contrast to the increasing trends observed in the other 20 regions.

Population growth, aging, and epidemiological change are all important contributors to the increased prevalence of prostate cancer globally, with population growth having the greatest impact (Figure 4 and Supplementary Table S2). Specifically, on a global scale, population growth, population aging, and epidemiological changes account for 65.62%, 17.97%, and 16.41%, respectively, of the increase in prostate cancer prevalence (Supplementary Table S2). When broken down into the five different SDI regions, this situation changes. Specifically, population growth plays a dominant role in high-SDI regions (account for 80.47%) and low SDI region (account for 64.11%), while epidemiological change is the primary factor in the other three SDI regions (high-middle SDI region account for 79.3%, middle SDI region account for 67.94% and low-middle SDI region account for 58.19%). It is evident that the global growth trend aligns with the trend in high-SDI regions, as more than half of the global prevalence is concentrated in these high-SDI areas. Additionally, decomposition analysis also reveals that the decline in high-income North America is primarily driven by epidemiological change, which serves as an excellent example of effective disease prevention.

Additionally, among these three driving factors, aging has become an increasingly significant factor that cannot be overlooked. Our APC analysis indicates that men over 50 years old have a higher risk of prostate cancer prevalence compared to population, with men aged 60–64 experiencing a six-fold higher risk compared to population. Globally, we will see a tripling in the number of people aged 60 years or older, increasing from 606 million today to nearly 2 billion by 2050, which highlights the growing aging population worldwide (55). Between 2020 and 2050, the worldwide population of people aged 60 and over will double to 2.1 billion, and the number of people aged 80 and over will triple to 426 million (56). In response to the increasing incidence of prostate cancer, particularly due to aging populations, public health organizations worldwide have updated their guidelines to recommend prostate-specific antigen (PSA)-based screening for men aged 75 years and older. This strategy aims to improve early detection and treatment outcomes (2). The World Health Organization (WHO) has also been advocating for increased awareness and prevention strategies to reduce the public health burden of prostate cancer in aging populations (4).

Quantifying cross-country inequalities in prostate cancer burden across the SDI gradient can help clarify the distribution pattern of the burden and identify countries where prostate cancer prevention and control need improvement. In this study, both absolute measure SII and relative measure CI revealed significant cross-country inequalities, with this imbalance progressively intensifying over time. This clustering phenomenon in high SDI regions helps explain the higher prostate cancer prevalence observed in these areas, with the United States contributing approximately one-quarter of the global prostate cancer prevalence. In response, the U.S. has implemented various healthcare strategies to manage this growing burden. These include the promotion of PSA-based screening for early detection, particularly for men aged 75 and older (2). Furthermore, advances in targeted therapies and immunotherapy have significantly improved treatment outcomes, offering hope to patients with advanced stages of prostate cancer (57). America's medical innovations not only benefit domestic patients but also contribute to the global advancement of cancer treatment by sharing research findings and therapeutic strategies, helping other countries improve their prostate cancer care.

As the Chinese saying goes, “The best doctor treats the disease before it occurs,” after a detailed analysis of the past 32 years of prostate cancer prevalence, we have predicted its trends for the next 25 years. To achieve this, the NORDPRED package was employed, offering key advantages such as accurate statistical projections using age-period-cohort (APC) models, which are crucial for forecasting cancer incidence and mortality and essential for public health planning (58, 59). With the aid of this statistical package, prostate cancer prevalence from 2022 to 2046 was predicted, showing a steady increase in both case numbers and age-standardized rates (ASR). Combining the preceding analysis of disease burden with this forecast analysis, we recommend stratified and targeted public health strategies across SDI levels: in low-SDI countries, prioritize community-based early detection programs by eliminating out-of-pocket screening costs, deploying mobile screening units, and enhancing health education to reduce financial, geographic, and awareness barriers while ensuring PSA or digital rectal examination (DRE) coverage for high-risk populations, and establish monitoring and evaluation mechanisms for continuous optimization (60); in low-middle SDI countries, strengthen diagnostic referral pathways and basic treatment capacity by integrating primary care with specialist services and employing low-cost imaging and laboratory diagnostics to improve post-diagnosis management efficiency (17, 61); in middle-SDI countries, implement personalized risk assessment and multidisciplinary care networks by incorporating genetic polymorphism screening tools to optimize screening intervals and protocols while promoting results-oriented oversight and continuous improvement systems (17, 62); and in high-SDI countries, enhance survivorship care planning by expanding health promotion, secondary cancer screening, and psychosocial support frameworks, leveraging digital health management and interdisciplinary collaboration to improve survivors' quality of life and overall health outcomes (63–65).

Several limitations should be noted regarding the Global Burden of Disease (GBD) database. The GBD framework is subject to uncertainty arising from sampling variability and the selection and fitting of complex statistical models, which can introduce model-driven biases (66). Concurrently, underreporting and misclassification persist due to incomplete medical records and diagnostic gaps, while the SDI, despite its utility, cannot capture within-country disparities or non-linear socioeconomic transitions (67, 68). Future research should enhance uncertainty quantification via sensitivity analyses, integrate novel data sources such as electronic health records, and employ scenario-based and machine learning approaches to improve projection reliability (69). Fortunately, the use of data cleaning, correction, and advanced statistical modeling methods by GBD collaborators effectively mitigates these limitations, enhancing the accuracy of health data. Despite these limitations, the study offers several key advantages. Through comprehensive analyses—including descriptive, trend, decomposition, health inequality, and predictive assessments—it enhances our understanding of prostate cancer epidemiology and provides valuable insights for shaping public health strategies and optimizing medical resource distribution globally.

## Conclusion

As a major health concern among older adult men, the global prevalence of prostate cancer has consistently increased from 1990 to 2021, with population growth being identified as the primary driver. Furthermore, cross-country inequalities highlighted significant SDI-related disparities across 204 countries and territories, with these inequalities becoming more pronounced over time. Finally, the APC model predicts a continuous rise in the prevalence of prostate cancer over the next 25 years, which highlights the substantial disease burden of prostate cancer and emphasizes the urgent need for more targeted and effective management strategies.

## Data Availability

The original contributions presented in the study are included in the article/Supplementary material, further inquiries can be directed to the corresponding authors.

## References

[1] Bergengren O Pekala KR Matsoukas K Fainberg J Mungovan SF Bratt O. 2022 update on prostate cancer epidemiology and risk factors-a systematic review. Eur Urol. (2023) 84:191–206. 10.1016/j.eururo.2023.04.021 37202314 PMC10851915

[2] Culp MB Soerjomataram I Efstathiou JA Bray F Jemal A. Recent global patterns in prostate cancer incidence and mortality rates. Eur Urol. (2020) 77:38–52. 10.1016/j.eururo.2019.08.005 31493960

[3] Sung H Ferlay J Siegel RL Laversanne M Soerjomataram I Jemal A. Global cancer statistics 2020: GLOBOCAN estimates of incidence and mortality worldwide for 36 cancers in 185 countries. CA Cancer J Clin. (2021) 71:209–49. 10.3322/caac.21660 33538338

[4] Rawla P. Epidemiology of prostate cancer. World J Oncol. (2019) 10:63–89. 10.14740/wjon1191 31068988 PMC6497009

[5] Appleton L Wyatt D Perkins E Parker C Crane J Jones A. The impact of prostate cancer on men's everyday life. Eur J Cancer Care. (2015) 24:71–84. 10.1111/ecc.12233 25204357

[6] Wasim S Lee SY Kim J. Complexities of prostate cancer. Int J Mol Sci. (2022) 23:14257. 10.3390/ijms232214257 36430730 PMC9696501

[7] Colloca G Colloca P. The effects of social support on health-related quality of life of patients with metastatic prostate cancer. J Cancer Educ. (2016) 31:244–52. 10.1007/s13187-015-0884-2 26174117

[8] Li Q Lin Y Xu Y Zhou H. The impact of depression and anxiety on quality of life in Chinese cancer patient-family caregiver dyads, a cross-sectional study. Health Qual Life Outcomes. (2018) 16:230. 10.1186/s12955-018-1051-3 30545383 PMC6293618

[9] Chen S Cao Z Prettner K Kuhn M Yang J Jiao L. Estimates and projections of the global economic cost of 29 cancers in 204 countries and territories from 2020 to 2050. JAMA Oncol. (2023) 9:465–72. 10.1001/jamaoncol.2022.7826 36821107 PMC9951101

[10] Hao S Östensson E Eklund M Grönberg H Nordström T Heintz E. The economic burden of prostate cancer – a Swedish prevalence-based register study. BMC Health Serv Res. (2020) 20:448. 10.1186/s12913-020-05265-8 32434566 PMC7238534

[11] Sangar VK Ragavan N Matanhelia SS Watson MW Blades RA. The economic consequences of prostate and bladder cancer in the UK. BJU Int. (2005) 95:59–63. 10.1111/j.1464-410X.2005.05249.x 15638895

[12] Bostwick DG Burke HB Djakiew D Euling S Ho SM Landolph J. Human prostate cancer risk factors. Cancer. (2004) 101:2371–490. 10.1002/cncr.20408 15495199

[13] Ferrís-i-Tortajada J García-i-Castell J Berbel-Tornero O Ortega-García JA. Constitutional risk factors in prostate cancer. Actas Urol Esp. (2011) 35:282–8. 10.1016/j.acuroe.2011.06.005 21435741

[14] GBD 2017 Diet Collaborators. Health effects of dietary risks in 195 countries, 1990-2017: a systematic analysis for the Global Burden of Disease study 2017. Lancet. (2019) 393:1958–72. 10.1016/S0140-6736(19)30041-8 30954305 PMC6899507

[15] Fathollahi F Khazaei Z Abbasi M Goodarzi E. Burden of prostate cancer and relationship with the Human Development Index (HDI) in Asia: a study of Global Burden Disease in 2019. Caspian J Intern Med. (2023) 14:710–9. 10.22088/cjim.14.4.710 38024182 PMC10646363

[16] Nowroozi A Roshani S Ghamari SH Shobeiri P Abbasi-Kangevari M Ebrahimi N. Global and regional quality of care index for prostate cancer: an analysis from the Global Burden of Disease study 1990-2019. Arch Public Health. (2023) 81:70. 10.1186/s13690-023-01087-2 37101304 PMC10131390

[17] Zhang W Cao G Wu F Wang Y Liu Z Hu H. Global burden of prostate cancer and association with socioeconomic status, 1990-2019: a systematic analysis from the Global Burden of Disease study. J Epidemiol Glob Health. (2023) 13:407–21. 10.1007/s44197-023-00103-6 37147513 PMC10469111

[18] Chu F Chen L Guan Q Chen Z Ji Q Ma Y. Global burden of prostate cancer: age-period-cohort analysis from 1990 to 2021 and projections until 2040. World J Surg Oncol. (2025) 23:98. 10.1186/s12957-025-03733-1 40114188 PMC11924780

[19] Scher HI Fizazi K Saad F Taplin ME Sternberg CN Miller K. Increased survival with enzalutamide in prostate cancer after chemotherapy. N Engl J Med. (2012) 367:1187–97. 10.1056/NEJMoa1207506 22894553

[20] Costello AJ. Considering the role of radical prostatectomy in 21st century prostate cancer care. Nat Rev Urol. (2020) 17:177–88. 10.1038/s41585-020-0287-y 32086498

[21] GBD 2019 Risk Factors Collaborators Ärnlöv J. Global burden of 87 risk factors in 204 countries and territories, 1990–2019: a systematic analysis for the Global Burden of Disease study 2019. Lancet. (2020) 396:1223–49. 10.1016/S0140-6736(20)30752-2 33069327 PMC7566194

[22] Cao F He Y-S Wang Y Zha C-K Lu J-M Tao L-M. Global burden and cross-country inequalities in autoimmune diseases from 1990 to 2019. Autoimmun Rev. (2023) 22:103326. 10.1016/j.autrev.2023.103326 36958621

[23] Liu Q He H Yang J Feng X Zhao F Lyu J. Changes in the global burden of depression from 1990 to 2017: findings from the Global Burden of Disease study. J Psychiatr Res. (2020) 126:134–40. 10.1016/j.jpsychires.2019.08.002 31439359

[24] Zhang D Liu S Li Z Wang R. Global, regional and national burden of gastroesophageal reflux disease, 1990–2019: update from the GBD 2019 study. Ann Med. (2022) 54:1372–84. 10.1080/07853890.2022.2074535 35579516 PMC9122392

[25] Goovaerts P. Analysis of geographical disparities in temporal trends of health outcomes using space–time joinpoint regression. Int J Appl Earth Obs Geoinf. (2013) 22:75–85. 10.1016/j.jag.2012.03.002 23710162 PMC3661294

[26] Bandi P Silver D Mijanovich T Macinko J. Temporal trends in motor vehicle fatalities in the United States, 1968 to 2010 - a joinpoint regression analysis. Inj Epidemiol. (2015) 2:4. 10.1186/s40621-015-0035-6 27747736 PMC5005740

[27] Kim HJ Fay MP Feuer EJ Midthune DN. Permutation tests for joinpoint regression with applications to cancer rates. Stat Med. (2000) 19:335–51. 10.1002/(sici)1097-0258(20000215)19:3<335::aid-sim336>3.0.co;2-z 10649300

[28] Welch WJ. Construction of permutation tests. J Am Stat Assoc. (1990) 85:693–8. 10.2307/2290004

[29] Henrique R Ribeiro FR Fonseca D Hoque MO Carvalho AL Costa VL. High promoter methylation levels of APC predict poor prognosis in sextant biopsies from prostate cancer patients. Clin Cancer Res. (2007) 13:6122–9. 10.1158/1078-0432.CCR-07-1042 17947477

[30] He H Liang L Han D Xu F Lyu J. Different trends in the incidence and mortality rates of prostate cancer between China and the USA: a joinpoint and age-period-cohort analysis. Front Med. (2022) 9:824464. 10.3389/fmed.2022.824464 35187007 PMC8850968

[31] Bray FI. Temporal Studies of Cancer Occurrence and Applications of the Age-Period-Cohort Method to Trends in Europe. London School of Hygiene & Tropical Medicine (2006).

[32] Chernyavskiy P Little MP Rosenberg PS. Correlated poisson models for age-period-cohort analysis. Stat Med. (2018) 37:405–24. 10.1002/sim.7519 28980325 PMC5768446

[33] Liu X Yu C Bi Y Zhang ZJ. Trends and age-period-cohort effect on incidence and mortality of prostate cancer from 1990 to 2017 in China. Public Health. (2019) 172:70–80. 10.1016/j.puhe.2019.04.016 31220754

[34] Theresa RS Jon W. A review and comparison of age–period–cohort models for cancer incidence. Stat Sci. (2016) 31:591–610. 10.1214/16-STS580

[35] Jackson JW VanderWeele TJ. Decomposition analysis to identify intervention targets for reducing disparities. Epidemiology. (2018) 29:825–35. 10.1097/EDE.0000000000000901 30063540 PMC6218173

[36] Cao F Xu Z Li XX Fu ZY Han RY Zhang JL. Trends and cross-country inequalities in the global burden of osteoarthritis, 1990-2019: a population-based study. Ageing Res Rev. (2024) 99:102382. 10.1016/j.arr.2024.102382 38917934

[37] Chen X Zheng J Wang J Wang H Shi H Jiang H. Global burden and cross-country inequalities in stroke and subtypes attributable to diet from 1990 to 2019. BMC Public Health. (2024) 24:1813. 10.1186/s12889-024-19337-5 38978043 PMC11229201

[38] Ni X Li Z Li X Zhang X Bai G Liu Y. Socioeconomic inequalities in cancer incidence and access to health services among children and adolescents in China: a cross-sectional study. Lancet. (2022) 400:1020–32. 10.1016/S0140-6736(22)01541-0 36154677

[39] Shan S Luo Z Yao L Zhou J Wu J Jiang D. Cross-country inequalities in disease burden and care quality of chronic kidney disease due to type 2 diabetes mellitus, 1990-2021: findings from the Global Burden of Disease study 2021. Diabetes Obes Metab. (2024) 26:5950–9. 10.1111/dom.15969 39344843

[40] Wang P Huang S Wang R Shi X Xu H Peng J. Global burden and cross-country inequalities in diseases associated with high body mass index from 1990 to 2019: result from the Global Burden of Disease study 2019. J Glob Health. (2024) 14:04200. 10.7189/jogh.14.04200 39513280 PMC11544517

[41] Wagstaff A Paci P van Doorslaer E. On the measurement of inequalities in health. Soc Sci Med. (1991) 33:545–57. 10.1016/0277-9536(91)90212-U 1962226

[42] Mackenbach JP Kunst AE. Measuring the magnitude of socio-economic inequalities in health: an overview of available measures illustrated with two examples from Europe. Soc Sci Med. (1997) 44:757–71. 10.1016/S0277-9536(96)00073-1 9080560

[43] Møller B Fekjær H Hakulinen T Sigvaldason H Storm HH Talbäck M. Prediction of cancer incidence in the Nordic countries: empirical comparison of different approaches. Stat Med. (2003) 22:2751–66. 10.1002/sim.1481 12939784

[44] Li D-P Han Y-X He Y-S Wen Y Liu Y-C Fu Z-Y. A global assessment of incidence trends of autoimmune diseases from 1990 to 2019 and predicted changes to 2040. Autoimmun Rev. (2023) 22:103407. 10.1016/j.autrev.2023.103407 37572826

[45] Berenguer CV Pereira F Câmara JS Pereira JAM. Underlying features of prostate cancer—statistics, risk factors, and emerging methods for its diagnosis. Curr Oncol. (2023) 30:2300–21. 10.3390/curroncol30020178 36826139 PMC9955741

[46] James LJ Wong G Craig JC Hanson CS Ju A Howard K. Men's perspectives of prostate cancer screening: a systematic review of qualitative studies. PLoS ONE. (2017) 12:e0188258. 10.1371/journal.pone.0188258 29182649 PMC5705146

[47] Gustavsen G Gullet L Cole D Lewine N Bishoff JT. Economic burden of illness associated with localized prostate cancer in the United States. Future Oncol. (2020) 16:4265–77. 10.2217/fon-2019-0639 31802704

[48] Stone BV Labban M Filipas DK Beatrici E Frego N Qian ZJ. Predictors of financial toxicity among United States prostate cancer survivors: results from a national survey. Urol Pract. (2023) 10:459–66. 10.1097/UPJ.0000000000000417 37498685

[49] Clements MB Vertosick EA Guerrios-Rivera L De Hoedt AM Hernandez J Liss MA. Defining the impact of family history on detection of high-grade prostate cancer in a large multi-institutional cohort. Eur Urol. (2022) 82:163–9. 10.1016/j.eururo.2021.12.011 34980493 PMC9243191

[50] Nyberg T Tischkowitz M Antoniou AC. BRCA1 and BRCA2 pathogenic variants and prostate cancer risk: systematic review and meta-analysis. Br J Cancer. (2022) 126:1067–81. 10.1038/s41416-021-01675-5 34963702 PMC8979955

[51] Siegel RL Miller KD Fuchs HE Jemal A. Cancer statistics, 2022. CA Cancer J Clin. (2022) 72:7–33. 10.3322/caac.21708 35020204

[52] Nicolosi P Ledet E Yang S Michalski S Freschi B O'Leary E. Prevalence of germline variants in prostate cancer and implications for current genetic testing guidelines. JAMA Oncol. (2019) 5:523–8. 10.1001/jamaoncol.2018.6760 30730552 PMC6459112

[53] Zhai Z Zheng Y Li N Deng Y Zhou L Tian T. Incidence and disease burden of prostate cancer from 1990 to 2017: results from the Global Burden of Disease study 2017. Cancer. (2020) 126:1969–78. 10.1002/cncr.32733 32012233

[54] Lin S Lin D Li Y Zhong L Zhou W Wu Y. Disease burden of prostate cancer from 2014 to 2019 in the United States: estimation from the Global Burden of Disease study 2019 and medical expenditure panel survey. Epidemiol Health. (2023) 45:e2023038. 10.4178/epih.e2023038 36996867 PMC10586921

[55] Hagmann M. The world in 2050: more crowded, urban and aged. Bull World Health Organ. (2001) 79:484. 11417047 PMC2566422

[56] Xi JY Lin X Hao YT. Measurement and projection of the burden of disease attributable to population aging in 188 countries, 1990-2050: a population-based study. J Glob Health. (2022) 12:04093. 10.7189/jogh.12.04093 36259226 PMC9579832

[57] Swami U McFarland TR Nussenzveig R Agarwal N. Advanced prostate cancer: treatment advances and future directions. Trends Cancer. (2020) 6:702–15. 10.1016/j.trecan.2020.04.010 32534790

[58] Sriplung H Singkham P Iamsirithaworn S Jiraphongsa C Bilheem S. Success of a cervical cancer screening program: trends in incidence in songkhla, southern Thailand, 1989-2010, and prediction of future incidences to 2030. Asian Pac J Cancer Prev. (2014) 15:10003–8. 10.7314/APJCP.2014.15.22.10003 25520060

[59] Yu XQ Luo Q Hughes S Wade S Caruana M Canfell K. Statistical projection methods for lung cancer incidence and mortality: a systematic review. BMJ Open. (2019) 9:e028497. 10.1136/bmjopen-2018-028497 31462469 PMC6720154

[60] Carneiro A Racy D Bacchi CE Leite KRM Filippi RZ Martins IAF. Consensus on screening, diagnosis, and staging tools for prostate cancer in developing countries: a report from the first prostate cancer consensus conference for developing countries (PCCCDC). JCO Glob Oncol. (2021) 7:516–22. 10.1200/GO.20.00527 33856895 PMC8162957

[61] Heijnsdijk EAM Gulati R Lange JM Tsodikov A Roberts R Etzioni R. Evaluation of prostate cancer screening strategies in a low-resource, high-risk population in the Bahamas. JAMA Health Forum. (2022) 3:e221116-e. 10.1001/jamahealthforum.2022.1116 35977253 PMC9123504

[62] Huynh-Le MP Karunamuni R Fan CC Asona L Thompson WK Martinez ME. Prostate cancer risk stratification improvement across multiple ancestries with new polygenic hazard score. Prostate Cancer Prostatic Dis. (2022) 25:755–61. 10.1038/s41391-022-00497-7 35152271 PMC9372232

[63] Resnick MJ Lacchetti C Bergman J Hauke RJ Hoffman KE Kungel TM. Prostate cancer survivorship care guideline: American Society of Clinical Oncology clinical practice guideline endorsement. J Clin Oncol. (2015) 33:1078–85. 10.1200/JCO.2014.60.2557 25667275

[64] Skolarus TA Wolf AM Erb NL Brooks DD Rivers BM Underwood W 3rd. American cancer society prostate cancer survivorship care guidelines. CA Cancer J Clin. (2014) 64:225–49. 10.3322/caac.21234 24916760

[65] Hart NH Nekhlyudov L Smith TJ Yee J Fitch MI Crawford GB. Survivorship care for people affected by advanced or metastatic cancer: MASCC-ASCO standards and practice recommendations. JCO Oncol Pract. (2024) 20:1160–72. 10.1200/OP.23.00716 38684036

[66] Vock DM Atchison EA Legler JM McClure DR Carlyle JC Jeavons EN. Accounting for model uncertainty in estimating Global Burden of Disease. Bull World Health Organ. (2011) 89:112–20. 10.2471/BLT.09.073577 21346922 PMC3040372

[67] von der Lippe E Devleesschauwer B Gourley M Haagsma J Hilderink H Porst M. Reflections on key methodological decisions in national burden of disease assessments. Arch Public Health. (2020) 78:137. 10.1186/s13690-020-00519-7 33384020 PMC7774238

[68] Murray CJL. The Global Burden of Disease study at 30 years. Nat Med. (2022) 28:2019–26. 10.1038/s41591-022-01990-1 36216939

[69] Muñoz Laguna J Puhan MA Rodríguez Artalejo F De Pauw R Wyper GMA Devleesschauwer B. Certainty of the Global Burden of Disease 2019 modelled prevalence estimates for musculoskeletal conditions: a meta-epidemiological study. Int J Public Health. (2023) 68:1605763. 10.3389/ijph.2023.1605763 37325175 PMC10266422

