# Supplementary material for: Global, regional, and national prevalence of prostate cancer from 1990 to 2021: a trend and health inequality analyses
Source: Front Public Health. 2025 Jun 11;13:1595159. doi: 10.3389/fpubh.2025.1595159 (PMC12187607; doi:10.3389/fpubh.2025.1595159)
Supplement: Supplementary file 2 [file Table_1.docx]

**Table S1. The case number and ASPR of prostate cancer in 1990 and 2021 and its temporal trends from 1990 to 2021 among 204 countries and territories.**

|  | **1990** | |  | **2021** | | **1990-2021 EAPC (95%UI)** | **1990-2021 Increased case number** |
| --- | --- | --- | --- | --- | --- | --- | --- |
| **Location** | **Case number (95% UI)** | **ASPR (95% UI)** |  | **Case number (95% UI)** | **ASPR (95% UI)** |  |  |
| Afghanistan | 1009 (709,1401) | 15.23 (11.07,20.75) |  | 1905 (1264,2580) | 23.07 (15.74,31.02) | 1.51 (1.27, 1.74) | 896 |
| Albania | 775 (598,1081) | 39.43 (30.64,55.23) |  | 3941 (2672,5777) | 83.83 (57.15,122.61) | 2.98 (2.67, 3.28) | 3166 |
| Algeria | 1328 (848,1748) | 11.59 (7.58,15.11) |  | 8548 (5595,12293) | 24.95 (16.42,35.52) | 2.70 (2.55, 2.85) | 7220 |
| American Samoa | 24 (19,32) | 118.39 (92.92,155.76) |  | 79 (61,104) | 172.66 (135.3,223.8) | 1.54 (1.36, 1.71) | 55 |
| Andorra | 144 (99,211) | 245.5 (169.86,356.08) |  | 537 (353,788) | 350.15 (228.72,514.57) | 1.43 (1.11, 1.75) | 393 |
| Angola | 832 (528,1122) | 25.46 (16.95,33.52) |  | 4716 (2897,6397) | 44.35 (27.68,59.27) | 1.90 (1.72, 2.07) | 3884 |
| Antigua and Barbuda | 181 (159,205) | 332.17 (291.71,375.78) |  | 558 (462,667) | 521.8 (435.69,622.27) | 1.09 (0.70, 1.48) | 377 |
| Argentina | 22924 (20501,25809) | 69.47 (62.29,77.86) |  | 63046 (54078,73755) | 110.73 (95.34,129.62) | 1.43 (0.97, 1.89) | 40122 |
| Armenia | 1032 (947,1129) | 36.43 (33.39,39.82) |  | 3981 (3355,4696) | 88.34 (74.99,103.74) | 3.52 (3.28, 3.75) | 2949 |
| Australia | 70208 (64127,74932) | 343.47 (314.21,366.76) |  | 200204 (170854,232687) | 434.99 (371.15,508.66) | 0.32 (-0.52, 1.16) | 129996 |
| Austria | 23193 (20496,26296) | 195.24 (171.58,221.29) |  | 52362 (43249,63299) | 299.69 (245.27,364.91) | 1.22 (0.72, 1.72) | 29169 |
| Azerbaijan | 1215 (909,1489) | 24.04 (18.24,29.25) |  | 4219 (3130,5667) | 40.01 (29.94,53.2) | 2.13 (1.93, 2.32) | 3004 |
| Bahrain | 133 (102,175) | 85.27 (64.24,112.52) |  | 2016 (1222,3041) | 241.25 (157.98,344.72) | 3.34 (3.23, 3.45) | 1883 |
| Bangladesh | 4313 (2707,6031) | 9.93 (6.22,13.89) |  | 26235 (13814,47935) | 19.05 (10.18,34.54) | 1.95 (1.86, 2.04) | 21922 |
| Barbados | 726 (615,848) | 234.56 (201.03,273.74) |  | 2000 (1500,2591) | 368.99 (277.27,478.7) | 1.43 (1.27, 1.60) | 1274 |
| Belarus | 8022 (6648,9178) | 60.04 (50,68.72) |  | 35367 (26402,46706) | 211.76 (158.75,278) | 3.92 (3.55, 4.29) | 27345 |
| Belgium | 31082 (27424,34790) | 193.13 (171.19,216.33) |  | 57148 (48537,65975) | 245.71 (207.21,284.94) | 0.51 (0.00, 1.03) | 26066 |
| Belize | 85 (76,94) | 93.66 (83.35,103.34) |  | 595 (499,707) | 212.61 (179.63,251.83) | 2.12 (1.53, 2.71) | 510 |
| Benin | 458 (360,611) | 24.37 (19.19,32.51) |  | 2410 (1327,3387) | 50.38 (28.36,70.88) | 2.56 (2.47, 2.64) | 1952 |
| Bermuda | 211 (184,242) | 335.08 (293.89,385.48) |  | 962 (759,1206) | 680.35 (537.53,855.07) | 2.21 (1.83, 2.58) | 751 |
| Bhutan | 16 (9,24) | 7.11 (4.05,11.06) |  | 95 (54,173) | 16.19 (9.24,29.43) | 2.72 (2.69, 2.75) | 79 |
| Bolivia | 1416 (984,2006) | 47.12 (32.83,66.02) |  | 8513 (5409,13172) | 94.53 (60.29,145.28) | 2.29 (2.20, 2.38) | 7097 |
| Bosnia and Herzegovina | 1421 (1115,1913) | 36.18 (28.5,48.87) |  | 6399 (4249,8761) | 97.52 (65.03,132.88) | 4.28 (3.82, 4.74) | 4978 |
| Botswana | 277 (198,364) | 51.77 (38.05,67.27) |  | 1210 (818,1673) | 84.44 (57.98,113.63) | 1.65 (1.50, 1.80) | 933 |
| Brazil | 57319 (54171,60116) | 66.42 (62.81,69.58) |  | 303693 (284262,322017) | 120.25 (112.54,127.43) | 1.58 (1.18, 1.99) | 246374 |
| Brunei | 29 (23,38) | 32.92 (25.83,43.31) |  | 172 (134,225) | 56.22 (44.36,72.53) | 2.02 (1.77, 2.27) | 143 |
| Bulgaria | 6440 (5624,7302) | 49.57 (43.36,55.43) |  | 18295 (15251,21730) | 119.42 (99.51,142.64) | 3.01 (2.85, 3.17) | 11855 |
| Burkina Faso | 915 (677,1211) | 22.47 (16.8,29.57) |  | 3901 (2138,5527) | 44.85 (24.97,63.01) | 2.60 (2.48, 2.72) | 2986 |
| Burundi | 639 (302,923) | 28.89 (13.98,41.3) |  | 1936 (980,3024) | 42.6 (22.03,65.24) | 1.36 (1.27, 1.46) | 1297 |
| Cambodia | 551 (386,718) | 13.49 (9.66,17.39) |  | 3748 (2351,5268) | 31.2 (19.82,43.31) | 3.04 (2.91, 3.17) | 3197 |
| Cameroon | 1181 (898,1544) | 29.51 (22.77,38.83) |  | 7554 (3757,11610) | 64.62 (32.93,97.61) | 2.72 (2.57, 2.87) | 6373 |
| Canada | 86105 (75306,97103) | 257.29 (225.31,292.02) |  | 157516 (136182,183806) | 215 (185.6,252.12) | -1.37 (-1.77, -0.97) | 71411 |
| Cape Verde | 97 (66,175) | 41.22 (27.65,74.26) |  | 672 (429,1054) | 156.56 (100.69,247.86) | 4.23 (3.97, 4.50) | 575 |
| Central African Republic | 215 (134,308) | 21.71 (14.24,29.85) |  | 498 (275,746) | 25.53 (14.85,37.01) | 0.59 (0.54, 0.65) | 283 |
| Chad | 505 (338,695) | 18.49 (12.45,25.26) |  | 2392 (1304,3620) | 45.39 (25.14,67.61) | 3.32 (3.18, 3.45) | 1887 |
| Chile | 7283 (6435,8337) | 74.29 (65.91,84.9) |  | 45149 (38104,53478) | 172.48 (145.64,204.38) | 2.92 (2.56, 3.28) | 37866 |
| China | 72323 (53379,90342) | 8.84 (6.63,11) |  | 627647 (450628,852641) | 28.51 (20.48,38.5) | 3.91 (3.78, 4.05) | 555324 |
| Colombia | 21719 (18916,24051) | 130.81 (114.1,144.75) |  | 149865 (119069,184131) | 272.97 (217.15,335.09) | 1.52 (1.13, 1.90) | 128146 |
| Comoros | 68 (32,102) | 36.27 (17.75,53.22) |  | 256 (143,384) | 54.37 (31.05,80.67) | 1.26 (1.18, 1.35) | 188 |
| Congo | 284 (169,450) | 29.56 (18.24,44.88) |  | 1392 (794,1929) | 57.09 (33.18,77.44) | 2.28 (2.06, 2.51) | 1108 |
| Cook Islands | 25 (19,33) | 208.56 (163.86,276.75) |  | 108 (84,143) | 400.99 (313.07,530.66) | 1.98 (1.75, 2.20) | 83 |
| Costa Rica | 2903 (2530,3283) | 173.17 (151.14,195.84) |  | 21044 (17784,25252) | 383.78 (324.89,459.3) | 2.56 (2.18, 2.93) | 18141 |
| Cote d'Ivoire | 2049 (1477,2682) | 60.23 (43.99,76.59) |  | 10149 (6454,14201) | 99.77 (65.67,136.84) | 1.60 (1.47, 1.72) | 8100 |
| Croatia | 5971 (5277,6706) | 99.3 (87.46,111.17) |  | 19608 (16232,23916) | 210.8 (174.85,255.89) | 2.96 (2.68, 3.23) | 13637 |
| Cuba | 22344 (19773,24902) | 214.5 (189.52,239.59) |  | 86032 (71194,102573) | 434.55 (357.85,520.98) | 2.30 (2.15, 2.44) | 63688 |
| Cyprus | 1267 (970,1665) | 150.4 (118.05,192.33) |  | 7183 (5405,10065) | 325.17 (243.78,456.4) | 3.05 (2.71, 3.39) | 5916 |
| Czech Republic | 12675 (11282,14159) | 88.4 (78.51,98.72) |  | 47553 (39011,56158) | 211.54 (171.84,251.06) | 2.83 (2.20, 3.46) | 34878 |
| Democratic Republic of the Congo | 3442 (2252,4565) | 26.12 (17.52,34.29) |  | 12834 (7977,18271) | 37.99 (23.99,53.31) | 1.27 (0.86, 1.69) | 9392 |
| Denmark | 11972 (10964,13710) | 140.05 (128.23,160.81) |  | 33990 (29489,38890) | 271.66 (234.05,310.82) | 3.22 (2.47, 3.98) | 22018 |
| Djibouti | 51 (24,82) | 42.35 (20.38,64.69) |  | 454 (231,727) | 75.05 (38.88,118.55) | 1.84 (1.75, 1.92) | 403 |
| Dominica | 106 (81,145) | 170.17 (129.79,233.6) |  | 240 (159,339) | 276.75 (185.13,389.53) | 1.28 (1.00, 1.56) | 134 |
| Dominican Republic | 3142 (2183,5266) | 90.03 (62.79,150.33) |  | 14672 (9182,24703) | 149.99 (93.73,252.59) | 1.49 (0.97, 2.01) | 11530 |
| Ecuador | 2745 (2462,3017) | 55.37 (49.76,60.84) |  | 18417 (13567,24191) | 112.79 (83.37,148.2) | 2.05 (1.51, 2.60) | 15672 |
| Egypt | 4112 (3259,5832) | 16.85 (13.46,24.27) |  | 37096 (22059,48990) | 63.06 (39.49,82.23) | 4.66 (4.43, 4.89) | 32984 |
| El Salvador | 2217 (1733,3479) | 77.46 (60.34,122.39) |  | 14085 (10375,19896) | 231.52 (169.92,327.28) | 3.31 (2.77, 3.84) | 11868 |
| Equatorial Guinea | 41 (25,58) | 23.63 (15.48,32.87) |  | 317 (170,476) | 69.16 (38.2,102.98) | 4.12 (3.93, 4.30) | 276 |
| Eritrea | 238 (106,362) | 23.14 (11.1,33.9) |  | 997 (506,1463) | 38.47 (20.05,54.75) | 1.41 (1.27, 1.54) | 759 |
| Estonia | 2356 (1952,2741) | 111.82 (92.95,129.52) |  | 11412 (8865,14480) | 427.92 (331.32,542.07) | 5.18 (4.46, 5.89) | 9056 |
| Ethiopia | 2011 (1233,2945) | 10.96 (6.91,15.85) |  | 7981 (4399,11910) | 19.92 (11.13,29.65) | 1.96 (1.82, 2.10) | 5970 |
| Federated States of Micronesia | 17 (13,24) | 36.32 (27.82,50.5) |  | 44 (30,59) | 62.98 (44.22,82.63) | 1.86 (1.80, 1.92) | 27 |
| Fiji | 118 (53,175) | 38.66 (17.78,56.07) |  | 358 (133,573) | 48.57 (18.76,77.06) | 0.84 (0.67, 1.00) | 240 |
| Finland | 11906 (10765,13231) | 159.06 (143.62,176.45) |  | 50099 (42380,58893) | 385.39 (323.24,457.78) | 2.49 (1.80, 3.18) | 38193 |
| France | 181767 (162448,200617) | 215.08 (191.74,237.86) |  | 487268 (405578,565454) | 361.88 (299.87,421.16) | 1.93 (1.61, 2.26) | 305501 |
| Gabon | 204 (126,305) | 36.73 (22.97,54.01) |  | 757 (402,1144) | 74.54 (40.26,110.65) | 2.40 (2.26, 2.55) | 553 |
| Georgia | 2768 (2503,3087) | 42.65 (38.68,47.45) |  | 6859 (5869,8016) | 113.88 (97.55,133.49) | 4.34 (3.82, 4.86) | 4091 |
| Germany | 203992 (185579,223389) | 155.2 (141.03,169.96) |  | 645444 (559284,739027) | 332.97 (286.46,382.16) | 2.09 (1.52, 2.68) | 441452 |
| Ghana | 2842 (1887,3807) | 50.34 (34.15,66.46) |  | 11817 (8807,16035) | 74.29 (55.04,99.57) | 0.98 (0.78, 1.18) | 8975 |
| Greece | 27624 (24362,31087) | 173.11 (152.81,194.33) |  | 49849 (43630,56642) | 212.9 (186.05,243.91) | 0.18 (-0.16, 0.52) | 22225 |
| Greenland | 15 (10,19) | 48.29 (32.61,59.28) |  | 54 (40,71) | 72.63 (55.14,93.45) | 1.76 (1.64, 1.88) | 39 |
| Grenada | 136 (117,158) | 190.77 (162.74,220.82) |  | 507 (421,600) | 427.65 (356.09,504.59) | 1.95 (1.13, 2.79) | 371 |
| Guam | 52 (42,74) | 74.09 (59.59,103.81) |  | 170 (135,229) | 79.77 (63.91,107.6) | 0.99 (0.62, 1.35) | 118 |
| Guatemala | 1425 (1295,1570) | 44.19 (40.43,48.6) |  | 15504 (12880,18844) | 144.03 (119.69,174.48) | 3.39 (2.51, 4.28) | 14079 |
| Guinea | 642 (473,836) | 20.23 (14.88,26.3) |  | 1864 (1210,2593) | 35.37 (23.13,48.9) | 1.93 (1.86, 2.00) | 1222 |
| Guinea-Bissau | 84 (58,133) | 23.53 (16.82,35.76) |  | 298 (155,460) | 45.5 (24.24,68.05) | 2.46 (2.34, 2.58) | 214 |
| Guyana | 383 (316,455) | 107.78 (89.86,127.41) |  | 1159 (849,1572) | 181.63 (135.22,242.43) | 1.52 (1.23, 1.82) | 776 |
| Haiti | 2225 (1606,2990) | 73.58 (53.72,97.01) |  | 7108 (4488,9729) | 109.47 (70.39,149.1) | 1.32 (1.14, 1.49) | 4883 |
| Honduras | 1005 (750,1378) | 52.12 (38.95,71.28) |  | 6401 (3912,10931) | 104 (63.28,177.6) | 2.39 (2.28, 2.50) | 5396 |
| Hungary | 10739 (9486,11992) | 69.68 (61.83,77.28) |  | 27471 (22398,33081) | 137.59 (111.1,165.58) | 1.96 (1.64, 2.28) | 16732 |
| Iceland | 836 (738,946) | 285.34 (252.13,323.04) |  | 2131 (1779,2549) | 358.48 (298.1,429.77) | 0.45 (0.11, 0.80) | 1295 |
| India | 33419 (22778,40866) | 7.82 (5.4,9.5) |  | 203775 (159791,268163) | 17.15 (13.49,22.48) | 2.24 (2.04, 2.44) | 170356 |
| Indonesia | 13914 (10009,16988) | 15.76 (11.58,19.07) |  | 86548 (54671,113864) | 37.92 (24.13,49.95) | 2.78 (2.72, 2.84) | 72634 |
| Iran | 10623 (7526,12998) | 41.16 (29.81,51.07) |  | 83364 (55106,100265) | 112 (75.11,134.63) | 3.45 (3.31, 3.59) | 72741 |
| Iraq | 1621 (1179,2338) | 21.9 (15.93,31.53) |  | 13791 (8958,19640) | 61.17 (40.23,87.09) | 3.77 (3.39, 4.14) | 12170 |
| Ireland | 8218 (7195,9215) | 188.29 (165.05,211.4) |  | 21323 (17967,25455) | 261.53 (219.23,312.15) | 1.49 (0.92, 2.06) | 13105 |
| Israel | 4803 (4266,5372) | 94.22 (83.76,105.43) |  | 15745 (13223,18662) | 125.2 (105.12,147.8) | 0.71 (0.12, 1.30) | 10942 |
| Italy | 159638 (145120,176643) | 172.38 (157.06,190.99) |  | 334843 (297440,373987) | 239.32 (211.09,268.32) | 0.95 (0.48, 1.43) | 175205 |
| Jamaica | 3301 (2905,3736) | 181.3 (159.25,205.6) |  | 12641 (9205,16684) | 417.8 (304.38,551.46) | 2.50 (1.69, 3.32) | 9340 |
| Japan | 74538 (70181,78765) | 43.64 (41.1,46.02) |  | 440834 (387463,485169) | 111.76 (98.56,123.47) | 3.39 (2.79, 3.99) | 366296 |
| Jordan | 470 (341,623) | 39.06 (28.36,51.16) |  | 6954 (4417,9770) | 98.53 (63.01,138.89) | 3.64 (3.33, 3.95) | 6484 |
| Kazakhstan | 3299 (2901,3708) | 25.71 (22.55,28.74) |  | 8417 (7208,9857) | 45.15 (38.72,52.92) | 2.83 (2.37, 3.28) | 5118 |
| Kenya | 1516 (920,2178) | 19.66 (12.11,28) |  | 8557 (5866,11227) | 37.97 (26.07,49.71) | 2.22 (2.09, 2.35) | 7041 |
| Kiribati | 4 (3,6) | 13.28 (10.15,16.86) |  | 12 (9,16) | 17.73 (13.2,23.77) | 0.94 (0.91, 0.98) | 8 |
| Kuwait | 321 (258,392) | 61.54 (50.18,74.14) |  | 5295 (3783,6925) | 215.48 (158.01,279.71) | 3.91 (3.22, 4.60) | 4974 |
| Kyrgyzstan | 647 (547,752) | 21.33 (18.08,24.44) |  | 1499 (1143,1913) | 31.25 (24.07,39.7) | 1.49 (1.16, 1.83) | 852 |
| Laos | 225 (152,311) | 11.58 (7.88,15.62) |  | 1035 (672,1457) | 24.25 (15.99,33.73) | 2.59 (2.51, 2.67) | 810 |
| Latvia | 2980 (2479,3418) | 81.32 (67.93,93.19) |  | 9437 (7219,11954) | 244.84 (185.93,312.58) | 4.15 (3.86, 4.44) | 6457 |
| Lebanon | 1490 (952,2177) | 70.12 (45.57,101.95) |  | 13304 (8987,18144) | 221.9 (150.08,305.17) | 4.47 (4.08, 4.87) | 11814 |
| Lesotho | 299 (212,410) | 36.03 (25.63,49.49) |  | 612 (406,922) | 55.27 (37.13,82.36) | 1.45 (1.28, 1.62) | 313 |
| Liberia | 266 (196,360) | 24.19 (18.15,32.34) |  | 1082 (544,1630) | 57.82 (29.86,86.09) | 3.20 (3.05, 3.35) | 816 |
| Libya | 1104 (661,1566) | 63.01 (37.87,89.81) |  | 5877 (3540,8213) | 124.68 (74.74,176.03) | 2.57 (2.26, 2.88) | 4773 |
| Lithuania | 6319 (4937,7546) | 138.75 (108.8,164.52) |  | 22930 (17940,29030) | 409.59 (317.07,521.16) | 3.94 (3.44, 4.44) | 16611 |
| Luxembourg | 860 (759,968) | 151.94 (134.13,171.11) |  | 2405 (2001,2844) | 228.3 (189.36,270.48) | 1.32 (0.98, 1.67) | 1545 |
| Macedonia | 809 (645,1106) | 45.11 (36.15,61.64) |  | 3484 (2371,4825) | 97.97 (67.81,134.35) | 3.13 (2.85, 3.42) | 2675 |
| Madagascar | 1671 (786,2434) | 34.93 (17.02,49.83) |  | 3918 (2106,6169) | 37.73 (20.86,57.77) | 0.22 (0.05, 0.40) | 2247 |
| Malawi | 771 (561,1032) | 21.55 (15.88,28.53) |  | 2917 (2100,3971) | 40.6 (29.39,54.33) | 2.25 (2.18, 2.32) | 2146 |
| Malaysia | 1992 (1424,2554) | 23.63 (16.78,30.16) |  | 15865 (10846,20583) | 56.74 (38.76,73.51) | 2.90 (2.76, 3.03) | 13873 |
| Maldives | 14 (10,19) | 18.91 (12.76,24.98) |  | 116 (80,158) | 39.63 (26.33,54.84) | 2.40 (2.13, 2.67) | 102 |
| Mali | 535 (396,665) | 15 (11.38,18.45) |  | 2014 (1404,2779) | 24.87 (17.82,34.02) | 1.78 (1.73, 1.82) | 1479 |
| Malta | 555 (477,653) | 126.49 (109.01,148.65) |  | 1863 (1529,2258) | 179.96 (147.39,219.24) | 1.01 (0.78, 1.25) | 1308 |
| Marshall Islands | 5 (4,7) | 33.13 (25.63,46.46) |  | 19 (12,26) | 62 (41.6,83.71) | 2.20 (2.11, 2.29) | 14 |
| Mauritania | 246 (180,343) | 25.51 (19,35.47) |  | 1761 (930,2581) | 84.94 (45.48,123.77) | 4.05 (3.97, 4.12) | 1515 |
| Mauritius | 214 (193,241) | 29.99 (27.14,33.54) |  | 1682 (1466,1906) | 87.85 (77.29,98.66) | 2.14 (1.71, 2.57) | 1468 |
| Mexico | 41781 (40176,43495) | 103.67 (99.58,107.83) |  | 227081 (189701,267738) | 181.51 (152.04,213.7) | 1.47 (1.12, 1.82) | 185300 |
| Moldova | 2157 (1889,2448) | 47.4 (41.63,53.68) |  | 8228 (6773,9800) | 132.61 (109.45,157.11) | 4.47 (3.81, 5.14) | 6071 |
| Mongolia | 61 (43,93) | 5.87 (4.25,8.88) |  | 308 (223,408) | 13.39 (9.82,17.74) | 3.54 (3.18, 3.89) | 247 |
| Montenegro | 694 (524,957) | 112.01 (84.65,155.56) |  | 2099 (1534,2884) | 199.95 (146.34,271.93) | 2.26 (1.90, 2.61) | 1405 |
| Morocco | 2149 (1338,2795) | 15.86 (9.94,20.6) |  | 13700 (7409,19105) | 40.17 (21.77,56.12) | 3.17 (2.85, 3.50) | 11551 |
| Mozambique | 576 (427,793) | 11 (8.41,14.57) |  | 1586 (1174,2437) | 15.4 (11.59,22.91) | 1.13 (1.08, 1.18) | 1010 |
| Myanmar | 2694 (1844,3619) | 12.49 (8.78,16.55) |  | 12643 (8285,17971) | 26.89 (17.69,37.87) | 2.67 (2.56, 2.78) | 9949 |
| Namibia | 229 (165,307) | 35.61 (25.89,46.92) |  | 1222 (745,1711) | 89.46 (55.12,123.29) | 3.62 (3.37, 3.87) | 993 |
| Nepal | 555 (355,819) | 6.59 (4.24,9.66) |  | 2962 (1820,5149) | 12.78 (7.96,22.07) | 2.18 (2.11, 2.25) | 2407 |
| Netherlands | 35394 (32459,38892) | 170.82 (156.68,187.3) |  | 101910 (88268,116133) | 279.01 (241.18,318.19) | 1.63 (1.17, 2.08) | 66516 |
| New Zealand | 15763 (13577,18185) | 387.35 (335.41,445.29) |  | 34937 (29636,41310) | 403.48 (341.78,477.8) | -0.25 (-0.47, -0.03) | 19174 |
| Nicaragua | 1104 (866,1561) | 76.71 (60.31,108.41) |  | 7531 (5383,10375) | 159.36 (114.18,219.28) | 2.50 (2.18, 2.83) | 6427 |
| Niger | 475 (319,679) | 19.43 (13.37,27.79) |  | 2886 (1456,4657) | 38.42 (20.09,60.94) | 2.61 (2.45, 2.78) | 2411 |
| Nigeria | 22936 (10886,33350) | 54.66 (26.63,78.57) |  | 77685 (32511,117378) | 94.77 (41.9,141.63) | 2.01 (1.88, 2.14) | 54749 |
| North Korea | 1420 (951,1902) | 9.28 (6.44,12.12) |  | 6428 (4547,8484) | 19.56 (13.98,25.61) | 2.90 (2.63, 3.17) | 5008 |
| Northern Mariana Islands | 11 (8,17) | 83.01 (60.98,123.71) |  | 65 (50,83) | 133.41 (102.32,170.17) | 1.45 (1.17, 1.73) | 54 |
| Norway | 15579 (14665,16594) | 213.51 (200.32,228.48) |  | 33316 (29190,37617) | 327.12 (286.93,368.97) | 1.49 (0.87, 2.11) | 17737 |
| Oman | 148 (96,207) | 23.93 (15.68,33.51) |  | 1111 (775,1517) | 60.81 (42.09,83.15) | 3.16 (2.83, 3.49) | 963 |
| Pakistan | 8118 (5942,10959) | 15.26 (11.19,20.61) |  | 29824 (20785,41608) | 26.51 (18.68,36.78) | 1.75 (1.68, 1.81) | 21706 |
| Palestine | 555 (402,787) | 66.54 (49.18,93.44) |  | 3469 (2535,5085) | 143.15 (106.97,207.23) | 2.45 (2.33, 2.57) | 2914 |
| Panama | 2419 (2098,2780) | 168.22 (146.2,193) |  | 15140 (11495,19157) | 346.34 (262.48,438.58) | 2.02 (1.45, 2.58) | 12721 |
| Papua New Guinea | 456 (273,691) | 29.61 (18.51,44.45) |  | 1979 (1122,3012) | 46.07 (27.12,69.39) | 1.48 (1.43, 1.53) | 1523 |
| Paraguay | 1128 (853,1659) | 52.83 (40.04,77.51) |  | 7778 (4630,11902) | 136.68 (81.5,208.48) | 2.71 (2.35, 3.06) | 6650 |
| Peru | 6343 (4552,8505) | 56.57 (40.79,75.98) |  | 48722 (32560,72509) | 149.77 (100.24,222.99) | 3.17 (2.99, 3.35) | 42379 |
| Philippines | 8710 (6434,11428) | 32.6 (24.39,42.72) |  | 38824 (28564,52456) | 48.89 (36.21,66.25) | 1.10 (1.01, 1.19) | 30114 |
| Poland | 17826 (16629,19118) | 39.64 (36.98,42.4) |  | 100221 (87006,113949) | 133.87 (116.43,151.74) | 4.15 (3.78, 4.53) | 82395 |
| Portugal | 20863 (18533,23506) | 141.68 (126.25,159.41) |  | 63498 (54216,74656) | 270.56 (229.43,318.32) | 1.82 (1.45, 2.20) | 42635 |
| Principality of Monaco | 146 (109,195) | 200.01 (148.55,271.02) |  | 333 (250,461) | 336.91 (250.43,466.13) | 1.78 (1.58, 1.98) | 187 |
| Puerto Rico | 7845 (6934,8916) | 209.72 (185.85,238.32) |  | 20236 (15875,24795) | 280.86 (220.08,346.19) | 0.77 (0.50, 1.04) | 12391 |
| Qatar | 128 (92,175) | 152.71 (110.07,210.45) |  | 3017 (1830,4973) | 408.63 (256.53,638.32) | 3.95 (3.63, 4.28) | 2889 |
| Republic of Nauru | 2 (1,3) | 53.62 (38.94,80.45) |  | 3 (2,6) | 62.85 (39.16,102.43) | 0.47 (0.22, 0.72) | 1 |
| Republic of Niue | 1 (1,2) | 50.51 (38.34,68.94) |  | 2 (1,3) | 96.1 (64.22,136.12) | 2.04 (1.90, 2.18) | 1 |
| Republic of Palau | 8 (6,11) | 82.53 (61.47,116.13) |  | 26 (18,37) | 117.11 (84.48,167.24) | 0.92 (0.79, 1.06) | 18 |
| Republic of San Marino | 81 (62,108) | 219.69 (167.73,292.61) |  | 151 (103,234) | 203.19 (135.45,320.47) | 0.51 (0.05, 0.96) | 70 |
| Romania | 11374 (9689,13203) | 39.81 (34.02,45.74) |  | 53788 (42478,66972) | 140.94 (111.08,175.59) | 4.44 (4.24, 4.65) | 42414 |
| Russian Federation | 87987 (83849,91523) | 46.39 (44.22,48.26) |  | 423189 (369505,467969) | 168.24 (146.99,185.96) | 4.68 (4.38, 4.99) | 335202 |
| Rwanda | 835 (397,1171) | 31.28 (15.49,42.98) |  | 3180 (1675,4711) | 51.27 (27.54,74.46) | 1.79 (1.50, 2.08) | 2345 |
| Saint Kitts and Nevis | 68 (60,76) | 163.76 (146.02,181.36) |  | 292 (215,387) | 423.8 (321.48,549.07) | 3.37 (2.83, 3.92) | 224 |
| Saint Lucia | 199 (175,221) | 220.08 (195.49,244.84) |  | 956 (748,1179) | 396.99 (312.08,489.66) | 1.45 (1.08, 1.82) | 757 |
| Saint Vincent and the Grenadines | 150 (134,169) | 201.81 (179.3,226.32) |  | 589 (502,685) | 401.4 (343.85,465.91) | 1.89 (1.64, 2.13) | 439 |
| Samoa | 26 (15,37) | 32.3 (18.91,44.37) |  | 67 (37,95) | 48.6 (27.83,68.54) | 1.18 (1.12, 1.24) | 41 |
| Sao Tome and Principe | 15 (12,20) | 24.12 (18.64,31.11) |  | 59 (42,84) | 56.72 (40.84,80.39) | 2.90 (2.81, 2.99) | 44 |
| Saudi Arabia | 1556 (1024,2714) | 30.63 (20.54,52.38) |  | 15288 (9699,29283) | 97.97 (64.26,183.77) | 3.83 (3.73, 3.92) | 13732 |
| Senegal | 844 (651,1106) | 27.95 (21.83,36.54) |  | 4961 (2716,7045) | 67.09 (37.22,94.53) | 3.07 (2.93, 3.21) | 4117 |
| Serbia | 6260 (4642,8220) | 58 (42.26,76.52) |  | 21754 (15074,29115) | 123.69 (85.57,165.48) | 3.03 (2.79, 3.27) | 15494 |
| Seychelles | 49 (34,84) | 85.53 (58.86,146.82) |  | 249 (187,316) | 218.33 (163.91,276.33) | 3.15 (2.69, 3.60) | 200 |
| Sierra Leone | 464 (349,627) | 23.66 (17.87,31.86) |  | 1760 (950,2628) | 50 (27.39,73.73) | 2.69 (2.50, 2.89) | 1296 |
| Singapore | 690 (594,785) | 34.42 (29.81,39.11) |  | 8713 (7179,10403) | 102.62 (84.43,122.02) | 3.58 (3.23, 3.93) | 8023 |
| Slovakia | 4957 (4040,6415) | 80.44 (65.47,103.98) |  | 17544 (11879,23738) | 173.61 (117.94,235.57) | 2.88 (2.74, 3.01) | 12587 |
| Slovenia | 2088 (1792,2389) | 83.82 (72.34,95.86) |  | 11004 (8714,13902) | 252.66 (198.82,319.97) | 4.21 (3.64, 4.79) | 8916 |
| Solomon Islands | 38 (24,54) | 32.69 (21.84,47.35) |  | 141 (96,200) | 46.81 (32.33,66.3) | 1.14 (1.02, 1.25) | 103 |
| Somalia | 524 (244,830) | 26.72 (12.99,41.14) |  | 1461 (727,2389) | 26.07 (13.55,41.39) | -0.01 (-0.03, 0.01) | 937 |
| South Africa | 11392 (7574,16024) | 57.91 (38.8,80.69) |  | 49302 (38455,59991) | 105.91 (82.56,128.77) | 2.22 (2.04, 2.39) | 37910 |
| South Korea | 3767 (2964,5877) | 14.22 (11.12,22) |  | 84728 (54336,112904) | 87.36 (55.91,116.45) | 6.57 (5.85, 7.30) | 80961 |
| South Sudan | 1043 (505,1579) | 40.86 (20.07,60.83) |  | 1734 (905,2683) | 49.72 (26.76,75.83) | 0.51 (0.46, 0.57) | 691 |
| Spain | 66733 (60699,73630) | 115.81 (105.19,127.67) |  | 189709 (163051,220580) | 199.92 (170.72,234.5) | 1.54 (1.00, 2.07) | 122976 |
| Sri Lanka | 1822 (1377,2400) | 18.13 (13.98,23.79) |  | 11042 (6973,16054) | 38.79 (24.73,55.79) | 2.72 (2.60, 2.84) | 9220 |
| Sudan | 1664 (1230,2207) | 18.61 (14.05,24.58) |  | 8796 (5762,12802) | 48.34 (32.53,70.27) | 3.23 (3.11, 3.36) | 7132 |
| Suriname | 243 (183,330) | 100.82 (76.11,137.95) |  | 954 (609,1422) | 150.76 (96.33,224.26) | 1.52 (1.30, 1.74) | 711 |
| Swaziland | 133 (94,172) | 49.54 (36,64.09) |  | 404 (236,619) | 72 (43.55,109.35) | 1.24 (1.15, 1.33) | 271 |
| Sweden | 43726 (38808,48360) | 278.3 (246.48,310.55) |  | 69279 (57965,82085) | 312.17 (259.55,371.31) | 0.50 (-0.03, 1.03) | 25553 |
| Switzerland | 32818 (29197,37592) | 303.49 (268.4,348.9) |  | 58098 (47890,69426) | 319.49 (260.71,383.6) | 0.05 (-0.31, 0.41) | 25280 |
| Syria | 1963 (1403,2736) | 39.69 (28.48,56.28) |  | 13932 (9500,21473) | 105.42 (72.64,161.56) | 3.09 (2.82, 3.37) | 11969 |
| Taiwan (Province of China) | 5953 (5447,6521) | 38.3 (35.11,42.02) |  | 58388 (50662,67517) | 134.23 (116.62,155.25) | 3.71 (3.03, 4.40) | 52435 |
| Tajikistan | 374 (273,484) | 13.83 (10.19,17.72) |  | 841 (565,1292) | 14.24 (9.84,20.88) | 0.04 (-0.12, 0.20) | 467 |
| Tanzania | 4300 (2010,6499) | 40.59 (19.5,60.34) |  | 13643 (7062,19983) | 56.14 (29.28,81.38) | 0.89 (0.80, 0.98) | 9343 |
| Thailand | 9735 (6167,13625) | 29.3 (19.03,40.75) |  | 85638 (45918,125369) | 77.32 (41.68,112.91) | 3.22 (3.02, 3.42) | 75903 |
| The Bahamas | 377 (326,428) | 253.57 (220.33,287.65) |  | 1559 (1245,1939) | 392.41 (314.5,487.86) | 1.50 (1.17, 1.83) | 1182 |
| The Gambia | 30 (22,39) | 9.36 (6.89,12.09) |  | 139 (93,190) | 15.29 (10.31,20.84) | 1.45 (1.33, 1.58) | 109 |
| Timor-Leste | 26 (15,39) | 11.73 (7,17.03) |  | 195 (129,272) | 22.85 (15.3,31.36) | 2.41 (2.28, 2.54) | 169 |
| Togo | 289 (219,386) | 26.52 (20.31,35.24) |  | 1999 (1020,2912) | 55.15 (28.84,79.1) | 2.41 (2.32, 2.49) | 1710 |
| Tokelau | 1 (0,1) | 48.93 (35.05,66.81) |  | 1 (1,2) | 94.62 (70.32,125.99) | 2.07 (2.02, 2.13) | 0 |
| Tonga | 42 (30,58) | 79.9 (57.06,108.11) |  | 91 (68,126) | 117.18 (87.65,160.89) | 1.12 (1.05, 1.19) | 49 |
| Trinidad and Tobago | 1752 (1572,1943) | 206.44 (185.41,228.61) |  | 6717 (4903,8922) | 332.36 (244.39,438.67) | 1.27 (1.09, 1.44) | 4965 |
| Tunisia | 1769 (1081,2488) | 35.88 (22.08,49.98) |  | 11111 (6544,17258) | 82.72 (49.19,127.62) | 2.75 (2.70, 2.80) | 9342 |
| Turkey | 19332 (13228,25248) | 59.15 (41.03,77.12) |  | 172324 (113280,241564) | 180.32 (118.84,250.64) | 3.82 (3.57, 4.06) | 152992 |
| Turkmenistan | 223 (199,249) | 11.54 (10.26,12.84) |  | 833 (642,1087) | 20.14 (15.64,26.15) | 2.64 (2.30, 2.99) | 610 |
| Tuvalu | 2 (1,3) | 28.05 (21.41,38.09) |  | 7 (5,9) | 62.65 (45.87,83.06) | 2.57 (2.40, 2.74) | 5 |
| Uganda | 3991 (2867,5149) | 64.88 (46.98,82.91) |  | 15114 (11026,20986) | 107.93 (79.34,147.95) | 1.51 (1.44, 1.59) | 11123 |
| Ukraine | 38150 (33705,42609) | 50.82 (45.12,56.41) |  | 60706 (40645,87294) | 75.39 (50.52,108.24) | 1.48 (1.32, 1.65) | 22556 |
| United Arab Emirates | 287 (200,435) | 76.68 (53.92,116.51) |  | 5692 (3880,8617) | 176.69 (127.61,261.48) | 3.84 (3.41, 4.27) | 5405 |
| United Kingdom | 163236 (157715,169087) | 171.02 (165.33,176.88) |  | 389168 (368402,406462) | 293.58 (278.61,306) | 1.88 (1.51, 2.24) | 225932 |
| United States | 1522355 (1465187,1576800) | 463.92 (447.23,479.53) |  | 2671779 (2547383,2786354) | 446.97 (425.84,465.84) | -0.45 (-0.56, -0.34) | 1149424 |
| Uruguay | 4575 (4057,5072) | 111.9 (99.06,124) |  | 10357 (8733,12095) | 186.85 (157.35,218.28) | 1.53 (1.08, 1.98) | 5782 |
| Uzbekistan | 1338 (1166,1510) | 11.74 (10.3,13.23) |  | 4273 (3454,5226) | 15.94 (13.03,19.11) | 1.33 (0.52, 2.15) | 2935 |
| Vanuatu | 19 (13,27) | 35.09 (25.42,49.18) |  | 73 (53,98) | 46.55 (34.39,61.93) | 0.93 (0.89, 0.98) | 54 |
| Venezuela | 13337 (11983,14705) | 144.89 (130.28,159.29) |  | 101589 (73144,135786) | 335.28 (243.2,447.83) | 2.18 (1.66, 2.71) | 88252 |
| Vietnam | 2325 (1459,3226) | 5.86 (3.71,8.09) |  | 16512 (9071,22835) | 17.1 (9.36,23.69) | 3.55 (3.43, 3.67) | 14187 |
| Virgin Islands, U.S. | 212 (150,293) | 260.13 (185.22,358.49) |  | 444 (253,785) | 222.35 (126.75,394.95) | -1.18 (-1.68, -0.67) | 232 |
| Yemen | 821 (543,1174) | 17.29 (11.44,24.63) |  | 5164 (3123,7424) | 39.01 (24.4,55.32) | 2.97 (2.77, 3.17) | 4343 |
| Zambia | 933 (469,1288) | 36.47 (18.85,49.75) |  | 6615 (2515,11127) | 98.74 (40.03,162.13) | 3.78 (3.15, 4.41) | 5682 |
| Zimbabwe | 2894 (2231,3732) | 74.36 (57.62,94.91) |  | 5811 (3589,7640) | 89.28 (55.75,116.48) | 0.39 (0.10, 0.69) | 2917 |

**Abbreviations:** ASPR, age-standardized prevalence rate; EAPC, estimated annual percentage change; UI, uncertainty interval; CI, conﬁdence interval.
