# Supplementary material for: Global, regional, and national prevalence of prostate cancer from 1990 to 2021: a trend and health inequality analyses
Source: Front Public Health. 2025 Jun 11;13:1595159. doi: 10.3389/fpubh.2025.1595159 (PMC12187607; doi:10.3389/fpubh.2025.1595159)
Supplement: Supplementary file 3 [file Table_2.docx]

**Table S2 Changes in prevalence of prostate cancer according to population-level determinants including aging, population growth and epidemiological change from 1990 to 2021**

| **Location** | **Overall difference** | **Aging** | **Population** | **Epidemiological change** | **Percent change of aging** | **Percent change of population** | **Percent change of epidemiological change** |
| --- | --- | --- | --- | --- | --- | --- | --- |
| Global | 18014310.8 | 2956786 | 11821237.12 | 3236288 | 16.41 | 65.62 | 17.97 |
| **SDI** |  |  |  |  |  |  |  |
| High SDI | 860482.85 | 376704.7 | 692391.545 | -208613 | 43.78 | 80.47 | -24.24 |
| High-middle SDI | 2319843.27 | 273567 | 206612.326 | 1839664 | 11.79 | 8.91 | 79.3 |
| Middle SDI | 3942218.59 | 349347.4 | 914675.557 | 2678196 | 8.86 | 23.2 | 67.94 |
| Low-middle SDI | 2631473.32 | 172309.9 | 927994.978 | 1531168 | 6.55 | 35.27 | 58.19 |
| Low SDI | 1222290.59 | 28255.4 | 783554.362 | 410480.8 | 2.31 | 64.11 | 33.58 |
| **Region** |  |  |  |  |  |  |  |
| Southeast Asia | 1070847 | 72442.62 | 274736.9 | 723667.4 | 6.76 | 25.66 | 67.58 |
| East Asia | 1232945 | 54627.23 | -73269.9 | 1251587 | 4.43 | -5.94 | 101.51 |
| Central Europe | 280032.7 | 3155.159 | -132274 | 409151.9 | 1.13 | -47.24 | 146.11 |
| Oceania | 23145.05 | 1015.757 | 15479.9 | 6649.396 | 4.39 | 66.88 | 28.73 |
| Central Asia | 82272.11 | 2840.194 | 34433.63 | 44998.28 | 3.45 | 41.85 | 54.69 |
| Eastern Europe | 568731.2 | -46908.6 | -200415 | 816054.4 | -8.25 | -35.24 | 143.49 |
| Western Europe | 1136824 | 7113.502 | -136238 | 1265948 | 0.63 | -11.98 | 111.36 |
| Australasia | 120993.7 | 11219.94 | 141246.4 | -31472.7 | 9.27 | 116.74 | -26.01 |
| Southern Latin America | 237362.3 | 20455.11 | 70372.6 | 146534.6 | 8.62 | 29.65 | 61.73 |
| High-income North America | -788133 | -73108.4 | 701957.5 | -1416982 | 9.28 | -89.07 | 179.79 |
| Andean Latin America | 307641.4 | 22446.31 | 107131.7 | 178063.4 | 7.3 | 34.82 | 57.88 |
| High-income Asia Pacific | 451409.5 | 95919.16 | -196441 | 551931.7 | 21.25 | -43.52 | 122.27 |
| Central Latin America | 1642546 | 183199.9 | 443652.6 | 1015694 | 11.15 | 27.01 | 61.84 |
| North Africa and Middle East | 2267843 | 171145.3 | 777917.9 | 1318780 | 7.55 | 34.3 | 58.15 |
| Caribbean | 346190.2 | 33733.59 | 84345.56 | 228111.1 | 9.74 | 24.36 | 65.89 |
| Tropical Latin America | 746154.5 | 87981.31 | 203867 | 454306.3 | 11.79 | 27.32 | 60.89 |
| South Asia | 1058431 | 88720.64 | 383861.9 | 585848.4 | 8.38 | 36.27 | 55.35 |
| Eastern Sub-Saharan Africa | 565195.9 | 22377.46 | 369790.5 | 173027.9 | 3.96 | 65.43 | 30.61 |
| Western Sub-Saharan Africa | 1291963 | -13539 | 845125.3 | 460377 | -1.05 | 65.41 | 35.63 |
| Central Sub-Saharan Africa | 225244.5 | 1048.429 | 153879.5 | 70316.56 | 0.47 | 68.32 | 31.22 |
| Southern Sub-Saharan Africa | 261975.6 | 24430.76 | 112800.9 | 124744 | 9.33 | 43.06 | 47.62 |

**SDI: socio-demographic index.**
