# Supplementary material for: Global, regional, and national prevalence of prostate cancer from 1990 to 2021: a trend and health inequality analyses
Source: Front Public Health. 2025 Jun 11;13:1595159. doi: 10.3389/fpubh.2025.1595159 (PMC12187607; doi:10.3389/fpubh.2025.1595159)
Supplement: Supplementary file 4 [file Table_3.docx]

**Table S3 The predicted ASR and case number of prevalence of prostate cancer between 2021 and 2046 at the global level.**

| **Year Group** | 2022-2026 | 2027-2031 | 2032-2036 | 2037-2041 | 2042-2046 |
| --- | --- | --- | --- | --- | --- |
| **Case number** | 11587678 | 13610965 | 15736691 | 17901343 | 20128150 |
| **ASPR** | 261.9298726 | 265.0851113 | 267.7820864 | 270.8344551 | 274.958302 |

**Abbreviations: ASPR,** age-standardized prevalence rate
