# Supplementary material for: Global, regional, and national prevalence of prostate cancer from 1990 to 2021: a trend and health inequality analyses
Source: Front Public Health. 2025 Jun 11;13:1595159. doi: 10.3389/fpubh.2025.1595159 (PMC12187607; doi:10.3389/fpubh.2025.1595159)
Supplement: Supplementary file 5 [file Table_4.docx]

**Table S4 The predicted ASR and case number of prevalence of prostate cancer between 2042 and 2046 among 192 countries and territories.**

| **Location** | **Number** | **ASPR** | **Location** | **Number** | **ASPR** | **Location** | **Number** | **ASPR** | **Location** | **Number** | **ASPR** |
| --- | --- | --- | --- | --- | --- | --- | --- | --- | --- | --- | --- |
| Afghanistan | 8538.880652 | 55.2598743 | Dominica | 287.4702972 | 514.8372601 | Lithuania | 37521.36831 | 1396.249825 | Sao Tome and Principe | 136.0859302 | 133.045361 |
| Albania | 5944.107307 | 196.3653817 | Dominican Republic | 32378.27886 | 374.5793192 | Luxembourg | 4472.315257 | 441.7852575 | Saudi Arabia | 53597.05665 | 276.0567193 |
| Algeria | 22908.38411 | 52.40116935 | Ecuador | 46419.85063 | 283.8173982 | Macedonia | 6473.952363 | 328.2433972 | Senegal | 29777.92147 | 357.4383654 |
| Andorra | 1182.920463 | 806.3730509 | Egypt | 78909.87116 | 114.1807021 | Madagascar | 10502.97101 | 71.82483385 | Serbia | 23110.08768 | 320.2067955 |
| Angola | 12921.30604 | 95.31277816 | El Salvador | 32759.32387 | 882.5423794 | Malawi | 9090.952073 | 114.7843685 | Seychelles | 687.4143288 | 516.4523363 |
| Antigua and Barbuda | 1186.9231 | 1188.314477 | Equatorial Guinea | 1012.454458 | 92.94223403 | Malaysia | 49175.4241 | 144.5274398 | Sierra Leone | 4762.994489 | 147.5664739 |
| Argentina | 119715.3393 | 283.832148 | Eritrea | 2221.138907 | 142.5611652 | Maldives | 506.2832083 | 83.01461091 | Singapore | 20773.3354 | 247.1327266 |
| Armenia | 6816.097896 | 300.1864683 | Estonia | 19615.31784 | 1278.756676 | Mali | 6025.028553 | 87.99131065 | Slovakia | 30822.46269 | 449.3081748 |
| Australia | 302511.5283 | 932.1800388 | Ethiopia | 21468.41039 | 40.03054922 | Malta | 3485.305933 | 432.0118525 | Slovenia | 17425.39298 | 625.1059706 |
| Austria | 70675.44533 | 618.4829161 | Federated States of Micronesia | 97.79716533 | 27.40221871 | Mauritania | 6129.78868 | 326.9866269 | Solomon Islands | 365.4807197 | 81.62684974 |
| Azerbaijan | 10427.51104 | 121.4287078 | Fiji | 551.391904 | 112.8846671 | Mauritius | 3067.884428 | 229.8930293 | Somalia | 4192.410275 | 61.04067109 |
| Bahrain | 5830.154518 | 610.9496301 | Finland | 62859.78413 | 856.2000424 | Mexico | 398034.4238 | 374.6714464 | South Africa | 125801.3715 | 337.3359186 |
| Bangladesh | 74903.5075 | 57.94474495 | France | 708749.7762 | 874.4713736 | Moldova | 9462.267951 | 465.8411274 | South Korea | 222809.8967 | 218.973496 |
| Barbados | 3184.966038 | 1039.349826 | Gabon | 1961.667484 | 141.0471901 | Mongolia | 1104.485383 | 48.29516542 | South Sudan | 4868.345266 | 100.7009976 |
| Belarus | 52476.61105 | 649.3944623 | Georgia | 9417.584161 | 315.8652656 | Montenegro | 3189.606279 | 520.5117081 | Spain | 316277.0585 | 437.2503596 |
| Belgium | 73720.99998 | 487.4564178 | Germany | 847885.4317 | 732.6226541 | Morocco | 35159.06449 | 108.2245398 | Sri Lanka | 22746.96919 | 103.5541573 |
| Belize | 1563.712894 | 537.6729323 | Ghana | 35485.19324 | 201.3793171 | Mozambique | 4730.600446 | 48.90624754 | Sudan | 21211.69564 | 105.4156294 |
| Benin | 7186.067655 | 129.208693 | Greece | 60349.2818 | 435.1594709 | Myanmar | 23636.82538 | 67.40201578 | Suriname | 3400.501022 | 684.668883 |
| Bermuda | 1452.041314 | 1445.170755 | Grenada | 1050.986489 | 1047.90218 | Namibia | 3284.790037 | 247.2231549 | Swaziland | 1065.171898 | 200.4896641 |
| Bhutan | 301.9761338 | 44.90348524 | Guam | 318.6427965 | 200.2531894 | Nepal | 7432.376771 | 46.25003 | Sweden | 87626.72775 | 646.1126249 |
| Bolivia | 16261.75219 | 221.8982298 | Guatemala | 31786.70993 | 317.7924568 | Netherlands | 146766.5966 | 623.0578437 | Switzerland | 82438.25133 | 633.2827869 |
| Bosnia and Herzegovina | 8434.675215 | 224.4749371 | Guinea-Bissau | 788.021647 | 89.99802312 | New Zealand | 54290.10353 | 876.2155703 | Syria | 41202.56948 | 272.6200559 |
| Botswana | 3253.976151 | 256.7671049 | Guinea | 5076.35499 | 99.57215155 | Nicaragua | 24325.34805 | 518.3183324 | Tajikistan | 2290.668729 | 40.00235012 |
| Brazil | 597902.3886 | 283.905643 | Guyana | 2059.97091 | 452.3241327 | Niger | 10862.83937 | 128.5957707 | Tanzania | 36538.38702 | 142.7261493 |
| Brunei | 585.0834272 | 124.0251484 | Haiti | 13611.26883 | 249.222989 | Nigeria | 189670.2424 | 233.1620669 | Thailand | 172344.3821 | 203.0326075 |
| Bulgaria | 19925.84667 | 263.2576567 | Honduras | 16426.52148 | 275.0468572 | North Korea | 14143.7942 | 53.14622868 | The Bahamas | 2772.346978 | 745.2760513 |
| Burkina Faso | 13077.45588 | 187.4944401 | Hungary | 32460.00324 | 307.2099136 | Norway | 52872.11978 | 688.1837513 | The Gambia | 339.7563451 | 30.58468768 |
| Burundi | 7242.355478 | 147.0480714 | Iceland | 3533.646909 | 683.6185761 | Oman | 2970.330381 | 123.4885128 | Timor-Leste | 530.7118928 | 79.57018697 |
| Cambodia | 11416.96658 | 103.4723347 | India | 469210.509 | 40.27711277 | Pakistan | 67380.84865 | 60.01707009 | Togo | 5133.087586 | 129.4407855 |
| Cameroon | 23163.33363 | 208.9932871 | Indonesia | 207345.2441 | 94.36308995 | Palestine | 11379.05838 | 401.3948797 | Tonga | 116.8783597 | 260.1093465 |
| Canada | 191448.0903 | 410.6576273 | Iran | 276610.5203 | 276.8896287 | Panama | 46352.59446 | 1008.496799 | Trinidad and Tobago | 12149.50361 | 806.4798319 |
| Cape Verde | 2210.446749 | 533.5436531 | Iraq | 45761.72235 | 202.9784221 | Papua New Guinea | 4387.46354 | 87.24772174 | Tunisia | 25789.14646 | 214.6013551 |
| Central African Republic | 576.2002415 | 58.26964767 | Ireland | 41271.84958 | 602.2673946 | Paraguay | 19221.58626 | 403.7613604 | Turkey | 425523.5497 | 479.0508459 |
| Chad | 7153.508444 | 124.7598085 | Israel | 25653.6213 | 287.2592489 | Peru | 141053.9171 | 436.4111924 | Turkmenistan | 2642.382205 | 58.2641286 |
| Chile | 145455.4292 | 573.0307304 | Italy | 434086.2689 | 503.130745 | Philippines | 82084.45246 | 123.1716521 | Uganda | 51747.15742 | 312.1782003 |
| China | 1396567.863 | 74.1137998 | Jamaica | 16484.30601 | 771.9298785 | Poland | 149861.3071 | 337.3033684 | Ukraine | 76489.26492 | 206.9457177 |
| Colombia | 288867.6384 | 518.8378859 | Japan | 561011.623 | 258.0630679 | Portugal | 81664.00478 | 597.9103208 | United Arab Emirates | 10230.33285 | 335.8747203 |
| Comoros | 465.320362 | 113.8160821 | Jordan | 28763.71845 | 313.5517931 | Principality of Monaco | 454.3789444 | 1139.683754 | United Kingdom | 620588.4822 | 706.2222913 |
| Congo | 3733.154566 | 135.1575958 | Kazakhstan | 18837.62408 | 126.17435 | Puerto Rico | 23810.29419 | 637.9834785 | United States | 3742356.741 | 943.7225632 |
| Costa Rica | 56364.01559 | 959.8597361 | Kenya | 31554.84207 | 134.8152787 | Qatar | 9199.726175 | 928.4349302 | Uruguay | 14000.32504 | 421.7352495 |
| Cote d'Ivoire | 31973.14377 | 243.3454718 | Kuwait | 9468.955485 | 354.1059854 | Republic of San Marino | 323.5452261 | 516.6214501 | Uzbekistan | 12740.07234 | 52.30950435 |
| Croatia | 25466.08301 | 525.8034417 | Kyrgyzstan | 3242.045105 | 75.91558113 | Romania | 77656.1114 | 371.2233662 | Vanuatu | 163.8523458 | 93.98909799 |
| Cuba | 145013.8725 | 1004.880483 | Laos | 3928.508547 | 88.73004964 | Russian Federation | 563942.1607 | 454.6358188 | Venezuela | 175027.4144 | 778.2907649 |
| Cyprus | 15659.09028 | 858.3481112 | Latvia | 14684.76219 | 820.9263043 | Rwanda | 7794.965991 | 129.1171297 | Vietnam | 44306.13911 | 47.7794209 |
| Czech Republic | 73116.74777 | 565.7117169 | Lebanon | 29265.32759 | 569.5211994 | Saint Kitts and Nevis | 518.0474973 | 1307.069063 | Virgin Islands, U.S. | 401.2094672 | 602.9706904 |
| Democratic Republic of the Congo | 33271.20982 | 86.79330629 | Lesotho | 1601.805033 | 190.7165362 | Saint Lucia | 1250.224839 | 808.2640794 | Yemen | 16915.95556 | 103.0888702 |
| Denmark | 50005.67909 | 622.6970726 | Liberia | 3282.499436 | 152.3272493 | Saint Vincent and the Grenadines | 749.631151 | 894.7764148 | Zambia | 33847.38493 | 417.9312394 |
| Djibouti | 1116.072493 | 156.5709997 | Libya | 26381.43252 | 415.1674558 | Samoa | 107.7557797 | 98.33192798 | Zimbabwe | 14014.97108 | 272.0777108 |

**Abbreviations: ASPR,** age-standardized prevalence rate
